# Artificial design of organic emitters *via* a genetic algorithm enhanced by a deep neural network†

**DOI:** 10.1039/d3sc05306g

**Published:** 2024-01-11

**Authors:** AkshatKumar Nigam, Robert Pollice, Pascal Friederich, Alán Aspuru-Guzik

**Affiliations:** a Chemical Physics Theory Group, Department of Chemistry, University of Toronto 80 St. George St Toronto Ontario M5S 3H6 Canada r.pollice@rug.nl aspuru@utoronto.ca; b Department of Computer Science, University of Toronto 40 St. George St Toronto Ontario M5S 2E4 Canada; c Institute of Nanotechnology, Karlsruhe Institute of Technology Hermann-von-Helmholtz-Platz 1 76344 Eggenstein-Leopoldshafen Germany; d Institute of Theoretical Informatics, Karlsruhe Institute of Technology Am Fasanengarten 5 76131 Karlsruhe Germany; e Vector Institute for Artificial Intelligence 661 University Ave Suite 710 Toronto Ontario M5G 1M1 Canada; f Department of Chemical Engineering & Applied Chemistry, University of Toronto 200 College St. Ontario M5S 3E5 Canada; g Department of Materials Science & Engineering, University of Toronto, 184 College St. Ontario M5S 3E4 Canada; h Lebovic Fellow, Canadian Institute for Advanced Research (CIFAR) 661 University Ave Toronto Ontario M5G Canada; i Acceleration Consortium Toronto Ontario M5G 3H6 Canada

## Abstract

The design of molecules requires multi-objective optimizations in high-dimensional chemical space with often conflicting target properties. To navigate this space, classical workflows rely on the domain knowledge and creativity of human experts, which can be the bottleneck in high-throughput approaches. Herein, we present an artificial molecular design workflow relying on a genetic algorithm and a deep neural network to find a new family of organic emitters with inverted singlet-triplet gaps and appreciable fluorescence rates. We combine high-throughput virtual screening and inverse design infused with domain knowledge and artificial intelligence to accelerate molecular generation significantly. This enabled us to explore more than 800 000 potential emitter molecules and find more than 10 000 candidates estimated to have inverted singlet-triplet gaps (INVEST) and appreciable fluorescence rates, many of which likely emit blue light. This class of molecules has the potential to realize a new generation of organic light-emitting diodes.

## Abstract 2

An artificial molecular design workflow using a genetic algorithm with molecular strings and artificial neural networks applied to organic molecules with inverted singlet-triplet gaps finds 1000 candidates with appreciable fluorescence rates.

## Graphical abstract

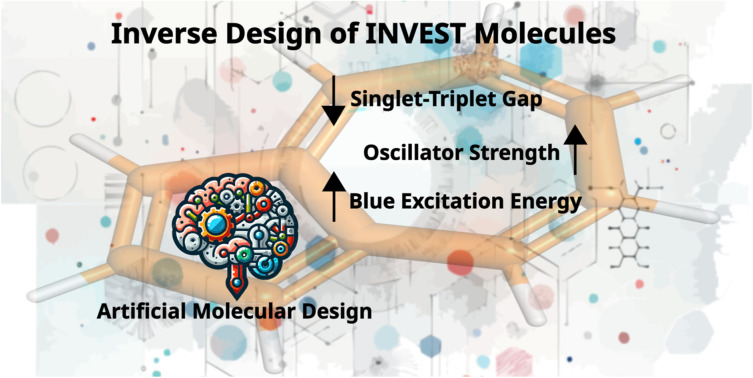

## Introduction

The introduction of SELFIES as a strictly robust molecular string representation not only allowed to enforce complete validity of every point in the latent space of deep generative models,^1^ but also enabled molecular generation *via* random string operations,^2^ which is an extremely inefficient process with the SMILES representation,^3^ as the overwhelming majority of random string modifications will lead to invalid SMILES strings. Accordingly, making use of SELFIES, the STONED algorithm allows for efficient and comprehensive navigation of the organic chemical space *via* random string modification and string interpolation.^2^ These unique capabilities of SELFIES can be leveraged in population-based metaheuristic optimization algorithms for inverse molecular design such as genetic algorithms^4–9^ (GAs) without relying on domain-specific genetic operators.^10,11^ Further enhancements of evolutionary algorithms *via* artificial neural networks (ANNs) have recently been demonstrated to improve molecular space exploration significantly leading to good performance in established artificial design benchmarks.^10–12^ Additionally, genetic algorithms for inverse molecular design showed consistently strong performance across multiple realistic molecular design domains in the Tartarus benchmarking suite.^12^ Specifically, genetic algorithms outperformed more sophisticated deep generative models such as variational autoencoders, sequence generation models, and flow-based generative models, without requiring any pre-training before initiating the inverse molecular design run. Thus, importantly, artificial molecular design workflows relying on genetic algorithms can be applied to any molecular design task with well-defined target properties out of the box even without prior knowledge of well-performing structural families.^13,14^ Furthermore, genetic algorithms are particularly suitable for target-oriented open ended design tasks as they explore the chemical space of interest as comprehensively as desired and they are not bound by the structure distribution of the training data.

State-of-the-art organic light-emitting diodes rely on molecules with energy differences below around 0.1 eV between the first excited singlet and the first excited triplet state,^15^ which enables efficient upconversion of non-emissive excited triplets to emissive excited singlets *via* reverse intersystem crossing in a mechanism referred to as thermally activated delayed fluorescence.^16^ While this mechanism enabled the realization of emissive devices with internal quantum efficiencies of 100%, long-term device performance can still suffer from substantial degradation caused by excited triplets, which are always present in substantial amounts, a problem particularly pronounced in blue organic emitters.^17,18^ In principle, this drawback could be overcome when molecules possess first excited singlet states that are lower in energy than the corresponding first excited triplet states. However, when Hund's first rule is applied to the first excited states, the first excited triplet state is predicted to be lower in energy compared to the corresponding singlet state.^19^ While Hund's first rule is not a fundamental law of physics, it provides an accurate description of the electronic structure of the vast majority of known molecules.^20^

Organic molecules with first excited singlet states lower in energy than the first excited triplet states are said to possess an inverted singlet-triplet gap (STG), which is referred to as INVEST.^21^ As they violate Hund's first rule, these molecules have been assumed to be extremely rare,^22,23^ however, recent work has uncovered a considerable number of structural families with that property,^21^ followed by systematic computational studies of their excited state properties.^24,25^ The inverted energy ordering between the first excited states stems from dynamic spin polarization stabilizing the first excited singlet relative to the triplet and this spin polarization is largely localized on a core structure.^26^ Hence, these core structures are responsible for the inverted energy gaps in all the known INVEST molecules, and recent experimental demonstrations have confirmed some of the predictions.^27^ Despite the promise of inverted STGs to increase device lifetimes in organic light-emitting diodes, most of the INVEST core structures found to date correlate with intrinsically low oscillator strengths (OSs) and, thus, slow fluorescence rates, which renders them ineffective as emitters. Accordingly, the design of organic emitters with both inverted STGs and appreciable OSs, resulting in high fluorescence rates, remains challenging and only a few studies relying on virtual screening of systematic datasets^28–30^ or with structure suggestions from human experts have been demonstrated.^21,26^

**Fig. 1:**
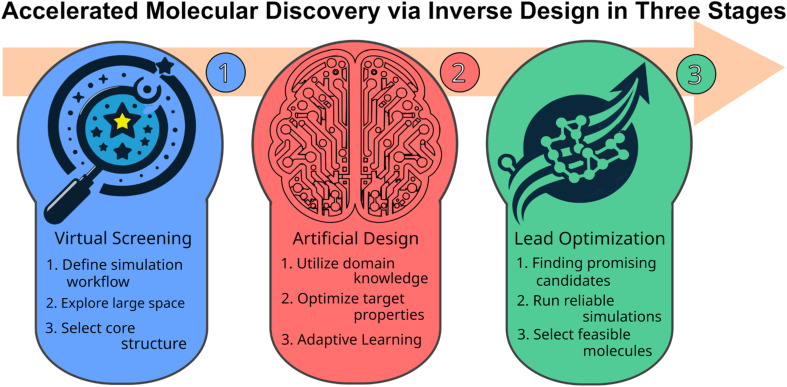
Accelerated molecular discovery workflow adopted in this work starting from high-throughput virtual screening, proceeding to artificial molecular design *via* a genetic algorithm enhanced by neural networks and filters based on domain knowledge, and finishing with lead validation.

In this work, we implement an artificial molecular design workflow to find organic INVEST emitters relying on a GA for efficient molecular generation making use of SELFIES and the STONED algorithm in the genetic operators. The complete workflow consists of hit identification *via* virtual screening, artificial molecular design and lead validation (Fig. 1). Sampling of the relevant molecular space is enhanced by a comprehensive set of filters based on domain knowledge and a data-driven ANN classifier that learns the structures of the best candidates encountered previously. This workflow relies on an efficient property simulation workflow for the relevant excited state properties implementing double-hybrid time-dependent density functional approximation (DH-TD-DFA) calculations. Thus, it enables us to explore more than 800 000 organic emitter candidates and uncover a new class of molecules with both inverted STG and appreciable OS possessing azulene core structures. More than 13 000 of the best candidates are evaluated with a reliable wavefunction-based excited state simulation method confirming that at least more than one thousand promising structures were uncovered, including potential blue emitters. Additionally, in the entire dataset, there are more than ten thousand molecules likely to have inverted STGs and appreciable OSs. Hence, this work expands the space of INVEST emitters dramatically and is the next step towards realizing the fifth generation of organic light-emitting diode materials.

## Results

### Virtual screening

We started this work by identifying promising new core structure families that both allow for the design of INVEST emitters with appreciable OS and are likely realizable in the laboratory. In a recent work, bottom-up construction rules for molecules with inverted STGs were established that facilitated the identification of 15 new core structure families predicted to have members being INVEST molecules.^26^ In that work, in addition to their excited state properties, their synthesizability and stability were assessed and one of the most promising core structures was proposed to be azulene due to the existence of reliable syntheses for a considerable range of derivatives.^26^ Azulenes are known to be very stable and are already widely used organic electronic materials.^31–35^ Based on that work, azulene was selected for further investigation.

However, we were still interested in identifying additional promising structures. Hence, by developing a comprehensive set of filters (*cf.* methods) we created a subset of GDB-13,^36^ originally comprising more than 970 million organic molecules, with over 400 000 structures possessing cycles and a high degree of conjugation. Subsequently, we performed high-throughput virtual screening of the corresponding structures relying on a quantum chemical DH-TD-DFA, namely ωB2PLYP’.^37^ This method has been benchmarked extensively against various reference methods that are based on excited state wavefunction theory approaches for simulating INVEST molecules and was shown to reproduce the property trends of INVEST molecules reliably.^21^ Additionally, based on these benchmarks, it provides the best trade-off between robustness and simulation cost, which is critical for high-throughput virtual screening. Notably, it is key to use computational methods that account for double excitations.^22^ Among the 292 structures with small predicted STGs below 0.25 eV, 61 structures (21%) were based on azulene, whereas 38 (13%) were based on pentalene, recently identified as INVEST motif using bottom-up construction rules,^26^ and only 11 (4%) on phenalene, which was studied extensively as core structure for INVEST emitters with appreciable fluorescence rates.^21^ Accordingly, azulene was again highlighted as promising INVEST core structure and we decided to focus our molecular design on this family for the rest of this work.

**Fig. 2:**
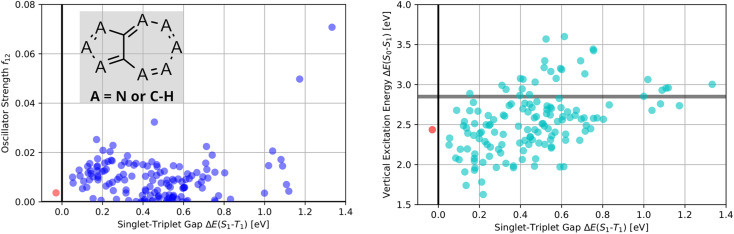
Property maps of all systematic permutations of nitrogen core structure substitutions of azulene at the EOM-CCSD/cc-pVDZ level of theory. (A) Singlet-triplet gap plotted against oscillator strength. (B) Singlet-triplet gap plotted against vertical excitation energy. The red data point denotes the only structure predicted to have an inverted singlet-triplet gap at this level of theory.

Thus, as established in a previous work on INVEST emitters based on phenalene cores,^21^ we generated all 144 systematic permutations of core structure nitrogen substitutions of azulene and simulated the corresponding excited state properties at the ωB2PLYP’,^37^ ADC(2),^38–44^ SOS-ADC(2)^41,45–53^ and EOM-CCSD^54–58^ levels of theory. The corresponding property maps at the EOM-CCSD level of theory are depicted in Fig. 2.

They reveal that only one of the nitrogen-substituted core structures, namely 2,5,7-triazaazulene (molecule 1, *cf.*Table 2), is predicted to have an inverted STG at that level of theory. Accordingly, we selected 1 as the starting point for our artificial design campaign described in the next section. Notably, the simulation results for all 144 azulene cores were compared to EOM-CCSD as reference method (*cf.* Supplementary Fig. 1 and Table 1†). The methods employed, while showing both deviations and uncertainties relative to EOM-CCSD, can reproduce trends in the three properties of interest, namely, STGs, OSs and vertical excitation energies (VEEs), and are, thus, appropriate for the subsequent artificial design workflow.^59^ Importantly, SOS-ADC(2) showed the most reliable property predictions compared to EOM-CCSD at only a fraction of the computational expense and, hence, it was decided to be used for the lead validation (*vide infra*).

### Artificial design

**Fig. 3:**
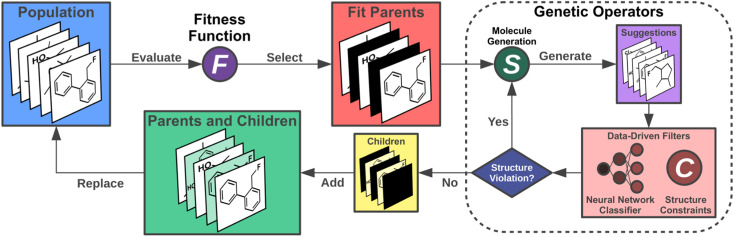
Artificial design workflow with a genetic algorithm employed for the design of organic INVEST emitters based on azulene enhanced by data-driven structure filters including an artificial neural network.

Having chosen the structural family to be investigated, next, we implemented the artificial design workflow (Fig. 3). We used a development version of JANUS,^11^ an extension of a previously published GA for inverse molecular design,^10^ that relies on the STONED algorithm^2^ for genetic operators but only propagates one generation of molecules. To evaluate the fitness of the proposed molecules, the excited state properties were simulated at the ωB2PLYP’ level of theory. The filters developed for the GDB-13 subset consisting of cyclic π-systems were implemented as necessary requirements for every structure generated, leading to increased sampling of the relevant structural space. Additionally, these filters were continuously updated based on expert opinion to eliminate infeasible structures proposed by our artificial design workflow. Furthermore, in each run, the first 11 generations were proposed without the use of ANNs enhancing sampling. Subsequently, all molecules encountered until generation 11 in each but the first experiment (*vide infra*) were used to train ANN classifiers identifying high-performing candidates at low computational cost and with high classification accuracy (*cf.*Table 1).

**Table 1:** Summary of the artificial design workflow results. Classification accuracy of the artificial neural network classifiers on both the validation and the holdout sets. Success rates of generating structures in the genetic operators with simulated singlet-triplet gaps below 0.6 and oscillator strength above 0.0 at the ωB2PLYP’ level of theory, both without and with the incorporation of the artificial neural network classifiers, in each experiment. Number of candidates generated in each run with predicted singlet-triplet gaps below 0.36 at the ωB2PLYP’ level of theory, which likely possess an inverted singlet-triplet gap, and number of candidates that additionally have a predicted oscillator strength above 0.05 at the ωB2PLYP’ level of theory. G: generation, STG: singlet-triplet gap, OS: oscillator strength, VEE: vertical excitation energy

Run	Classification accuracy		Success rate		Candidates	
	Validation	Holdout	G 11, without classifier	G 12, with classifier	STG < 0.36 eV	STG < 0.36 eV, OS > 0.05
1	—	—	—	—	809	2
2	92.0%	91.0%	7.8%	31.3%	25 503	312
3	98.0%	98.0%	7.0%	23.3%	24 142	293
4	91.0%	90.0%	7.5%	24.8%	27 867	334
5	89.0%	89.0%	6.6%	28.9%	34 235	6811
6	90.0%	89.0%	6.9%	27.1%	50 266	3074
All	—	—	—	—	148 311	10 736

These classifiers were incorporated into the genetic operators and used as additional filters. Hence, only molecules classified as good were passed on to the fitness evaluation to reduce the number of costly DFT simulations for 4 subsequent generations and improve the exploration of promising candidates even further. This is demonstrated based on the success rates of generating molecules with low singlet-triplet gaps (STGs) and non-zero OSs in each of the experiments which increased to 3-4 times the original value when the classifier was incorporated (*cf.*Table 1). Notably, as detailed below, we also explored the use of a few alternative fitness evaluation procedures. In all runs, structures with STGs above a certain threshold were assigned a very low fitness. Finally, to avoid prohibitively expensive quantum chemistry simulations, we capped the size of the molecules generated at 70 atoms, including hydrogens, and we only allowed previously unseen structures.

**Fig. 4:**
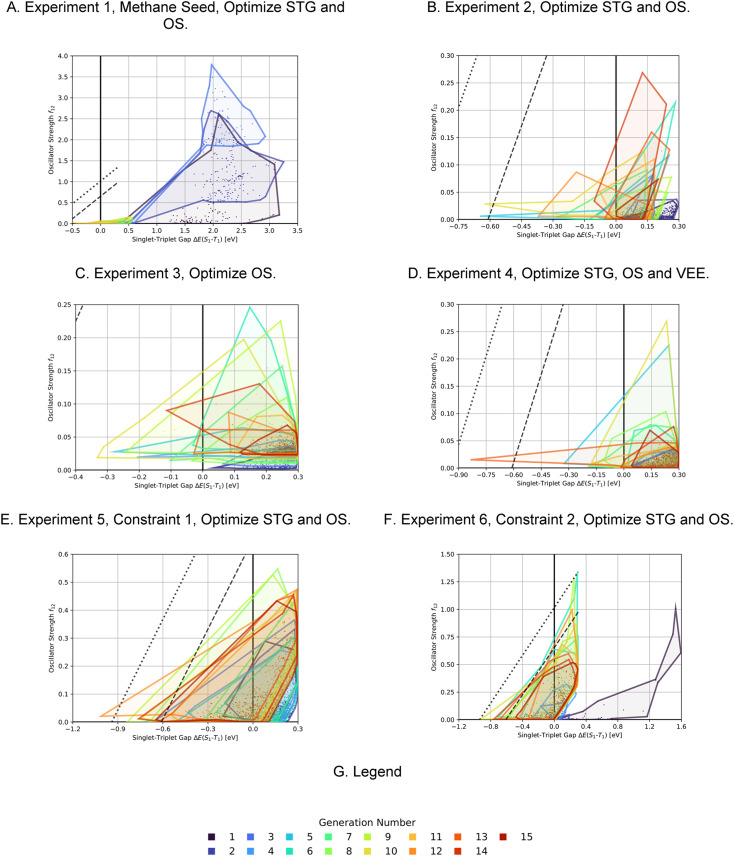
Progress of the property distributions spanned by the 200 molecules with highest fitness with respect to singlet-triplet gaps (STGs) and oscillator strengths (OSs) as a function of the generation numbers in each of the six artificial design experiments carried out (A)–(F) and the corresponding legend (G). The individual data points mark the properties of the molecules encountered, the enclosed areas of each generation are the corresponding alpha shapes of the point clouds. The dashed and dotted lines in each plot are at identical coordinates and are visual anchors indicating the edge of the property distribution reached in experiment 6.

As the first artificial design experiment, we used methane as seed molecule for the first generation and used OS minus STG as a fitness function, with an upper STG threshold of 0.6 eV for high fitness (see computational methods and Table 5 for details and mathematical expressions). We wanted to test our workflow for its ability to discover potential INVEST core structures without bias from the seed. The corresponding optimization progress is depicted in Fig. 4A. After three generations that explore the property space very extensively, the optimization trajectory focusses on promising candidates with low STGs and non-negligible OSs. Notably, in this run we did not train a classifier after 11 generations of experiments but stopped the study, as the goal of the experiment was to find potential interesting hits rather than perform comprehensive optimization. Indeed, azulenes were already explored in the first generation suggesting that the implemented filters strongly bias the molecular generation towards relevant cyclic π-systems. Apart from azulenes, several other known INVEST core structures were identified as promising candidates including cyclobuta-1,3-diene, cycloocta-1,3,5,7-tetraene, pentalene, bowtiene, heptalene, zurlene and anthrazulene.^26^ Importantly, azulenes accounted for 6% of all the structures explored and they were also most prevalent among the best candidates proposed in our first experiment. This reaffirmed our decision to focus all subsequent artificial design efforts on azulenes. Finally, while the best candidates possessed promising STGs, the OSs were only improved to a limited extent.

**Fig. 5:**
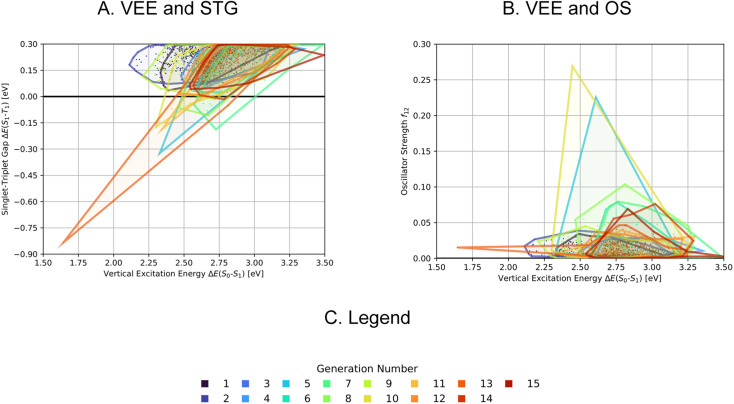
Progress of the property distributions spanned by the 200 molecules with highest fitness with respect to singlet-triplet gaps, oscillator strengths and vertical excitation energies as a function of the generation numbers in artificial design experiment 4 (A) and (B) and the corresponding legend (C). The individual data points mark the properties of the molecules encountered, the enclosed areas of each generation are the corresponding alpha shapes of the point clouds. STG: Singlet-triplet gap, OS: oscillator strength, VEE: vertical excitation energy.

**Fig. 6:**
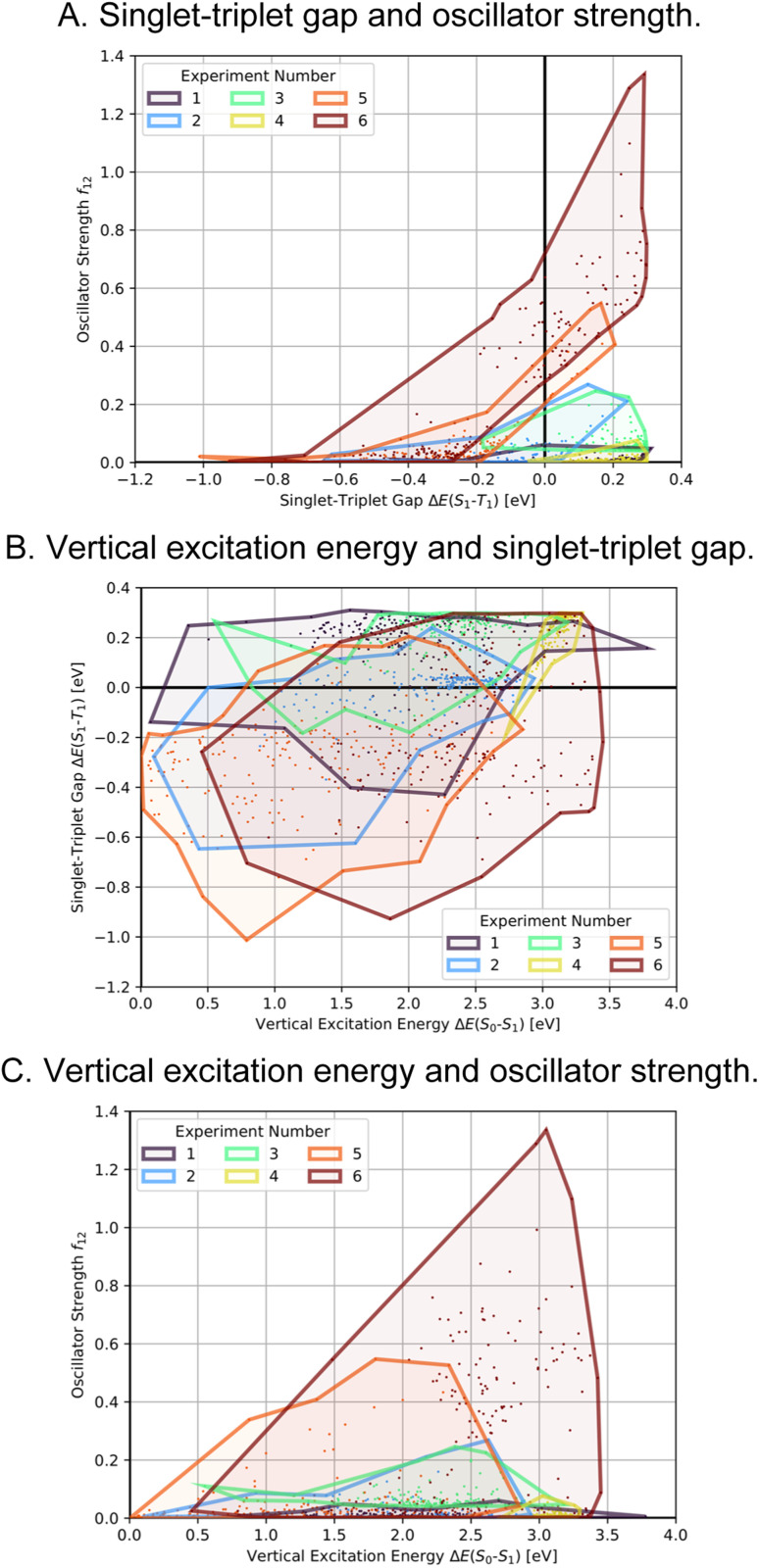
Comparison of the property distributions spanned by the 200 molecules with highest fitness proposed in each of the six artificial design experiments conducted (A)–(C). The individual data points mark the properties of the molecules encountered, the enclosed areas of each generation are the corresponding alpha shapes of the point clouds.

In the second, third and fourth artificial design experiments, we used molecule 1 as initial seed. Additionally, only structures containing azulene-like π-systems were accepted in the molecular generation to ensure extensive exploration of that structural family. Furthermore, the upper STG threshold for high fitness values was 0.3 eV in all these runs. The only difference between these three experiments was the fitness function employed. In experiment 2, as in experiment 1, a linear combination of the additive inverse of the STG and OS was used. In experiment 3, only the OS determined the fitness. In experiment 4, the fitness was a linear combination of the additive inverse of the STG, the OS and the absolute difference to a VEE of 3.2 eV. The latter value corresponds to the energy of blue light absorption, but only after correction for the inherent systematic offset of ωB2PLYP’.^21^ Again, optimization progresses are depicted in Fig. 4B–D. Most importantly, compared to the first run, both lower STGs and higher OSs are attained in all three runs resulting in promising INVEST emitter candidates (*cf.*Fig. 6A). When comparing experiments 2 and 3, we were surprised to see that including the STG explicitly into the fitness function does not seem to result in molecules with lower STGs. However, as we expected, experiment 3 results in property distributions biased towards higher OS values. Strikingly, experiment 4 resulted in candidates with both the highest OSs and the lowest STGs among the three runs discussed in this paragraph. Notably, the corresponding optimization progress with respect to the VEEs is depicted in Fig. 5 showing that the optimization trajectory moved continuously towards higher VEEs.

In order to test whether the OSs can be further increased without compromising the STGs, we analyzed the high performing molecules and noticed that several promising candidates had substituents both in the 1- and 6-positions of the azulene core (*cf.* Supplementary Fig. 2†). Hence, to narrow down the space to be explored, focus on more promising structures and increase synthesizability, we decided to not only constrain the molecules generated in experiment 5 to possess an azulene-like π-system, but also enforce them to be identically substituted at the 1- and 6-positions. This was achieved by first generating the structures of the substituents which were subsequently attached to an azulene core structure only at the respective positions. Additionally, we decided to again use a linear combination of the additive inverse of the STG and the OS as fitness function. The corresponding optimization progress and property distributions (*cf.*Fig. 4E and 6) confirmed that this design choice indeed resulted in significantly better candidates as both STGs tended to be lower and OSs tended to be higher.

Encouraged by the results of experiment 5, we wanted to increase the sampling of promising molecules even further and decided to enforce the structures to have a plane of symmetry through the azulene core. Additionally, we also kept the core nitrogen substitutions equivalent to molecule 1 in all proposed structures. Furthermore, we decided to only allow substitutions at the 4- and 8-positions as these would be preferred for the introduction of donor moieties based on the bottom-up design principles for INVEST emitters established previously.^26^ As evident from the results (*cf.*Fig. 4F and 6), this design space resulted in by far the best organic emitter candidates among all the six artificial design experiments carried out. While the STG distributions were essentially equivalent to experiment 5, the OSs made a significant leap, reaching values far larger than 1. Importantly, these are better property trade-offs than have been attained in previous expert-guided INVEST emitter designs.^21^ Additionally, even though the VEEs were not explicitly optimized in this run, a significant fraction of the structures generated in experiment 6 had VEEs in the blue light region. Furthermore, our artificial design workflow incorporated intramolecular hydrogen-bonding to the core nitrogen atoms in the most promising candidates, which has been proposed before as a very effective strategy to increase OSs of INVEST emitters.^21^

**Fig. 7:**
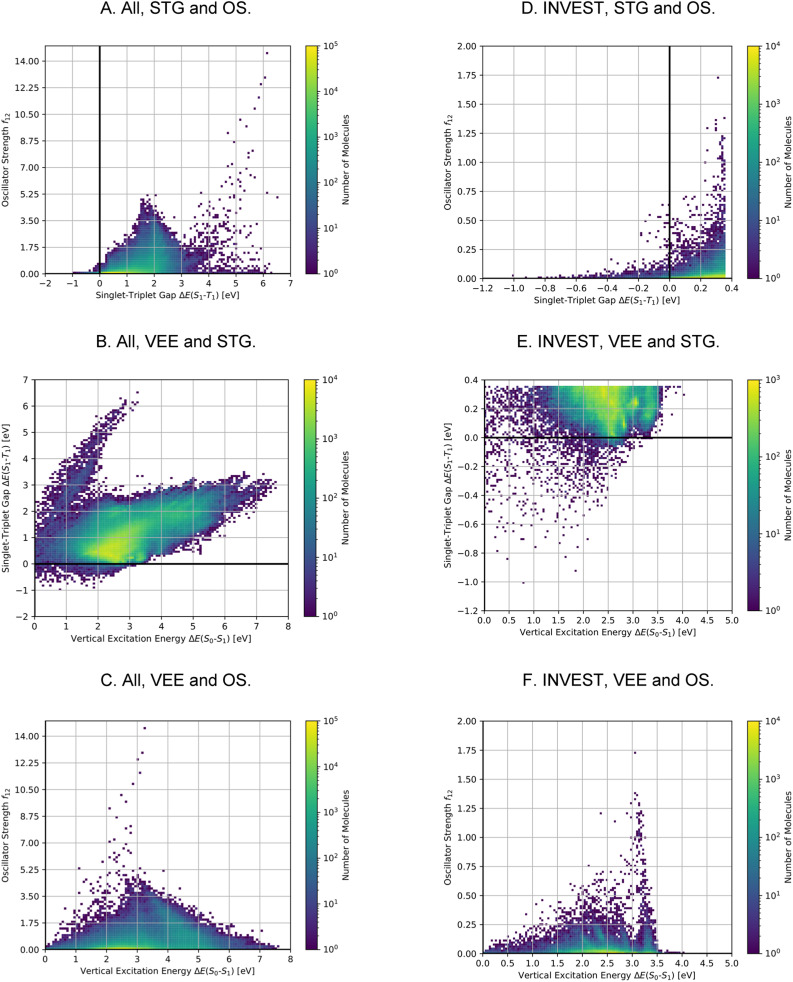
Property distributions of all the compounds generated during the artificial design stage (A)–(C) and the subset that is estimated to consist of INVEST compounds (D)–(F) at the ωB2PLYP’ level of theory colored by the number of molecules in the respective property windows. STG: Singlet-triplet gap, OS: oscillator strength, VEE: vertical excitation energy.

A comparison of the property distributions of the molecules with highest fitness in each experiment is depicted in Fig. 6. It suggests that, by altering the setups in each run, we successfully directed our artificial design workflow to ever more promising organic INVEST emitters. Additionally, in Table 2 we also compared some of the molecules with high fitness in each of the runs and their properties as this comparison provides an overview of the structural features characteristic of each artificial design experiment and of the diversity of structures generated. Importantly, all the molecules shown are likely stable and, thus, should in principle be realizable in the laboratory. A combined property distribution map of all the 869 365 molecules generated and simulated in the course of the artificial design experiments can be found in Fig. 7A–C. Individual property distribution maps for each experiment are depicted in Supplementary Fig. 3–8.†

**Table 2:** Comparison of the seed molecule 1 established in the virtual screening and some of the most promising candidates that emerged from each of the six artificial design experiments conducted. Excited state properties are at the ωB2PLYP’/def2-mSVP level of theory

Experiment	Molecule	Δ*E*(S_1_–T_1_) [eV]	*f*_12_	Δ*E*(S_0_–S_1_) [eV]
0 (seed)	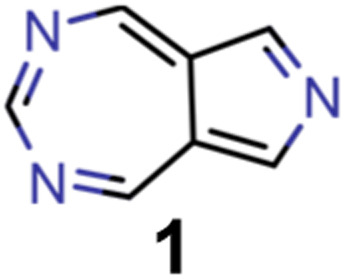	0.24	0.005	2.71
1	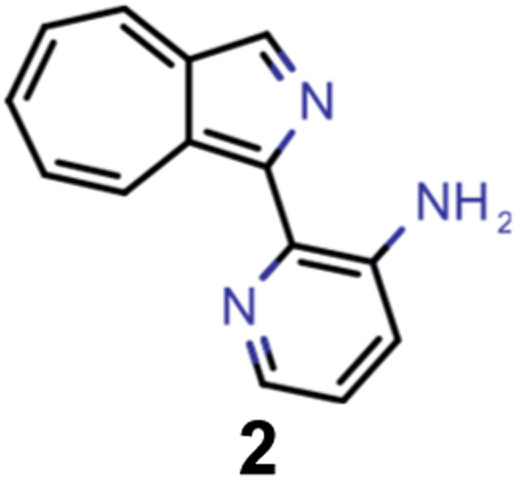	0.39	0.045	2.14
1	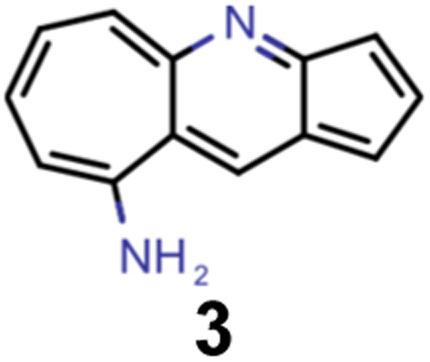	0.23	0.024	1.70
2	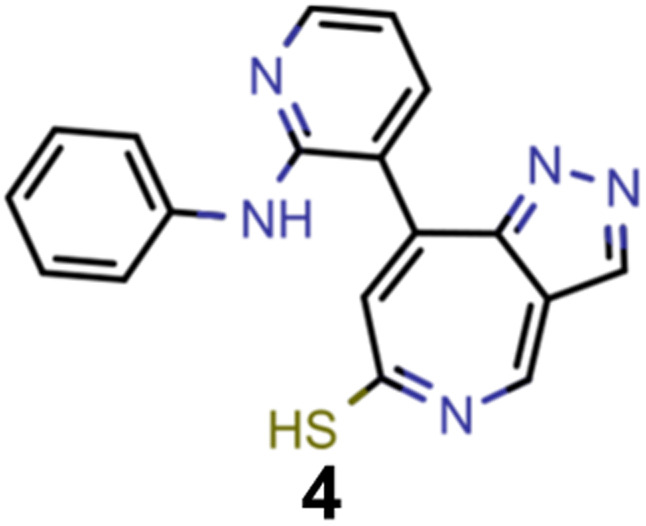	0.13	0.269	2.63
2	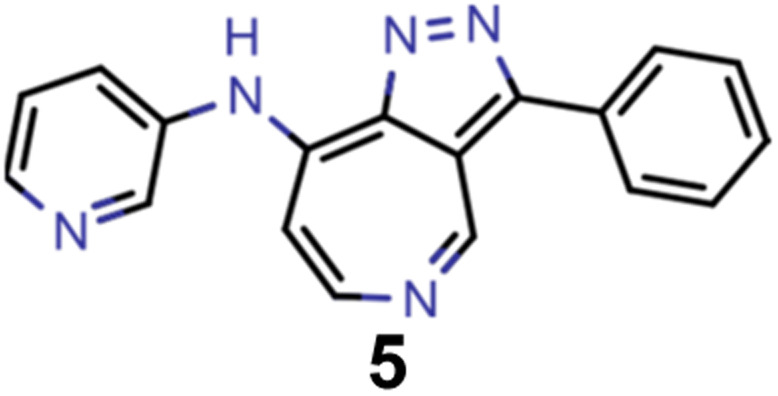	0.30	0.079	3.19
3	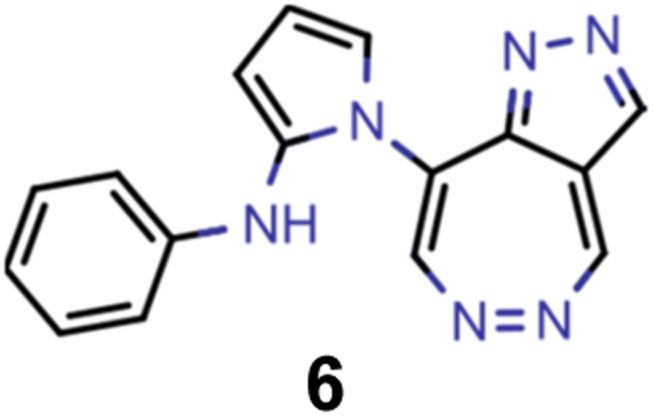	0.08	0.087	1.71
3	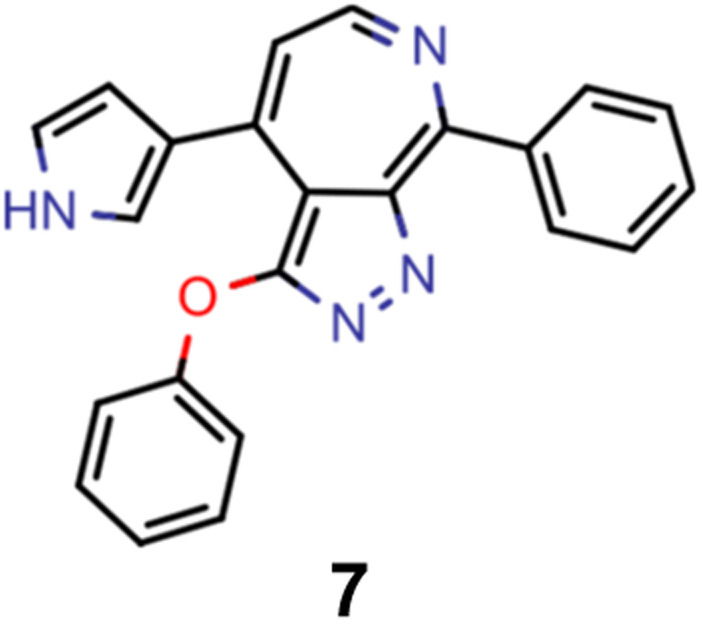	0.25	0.083	2.65
4	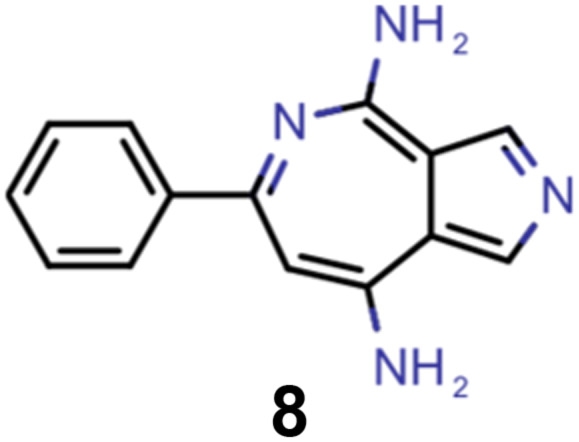	0.30	0.048	3.23
4	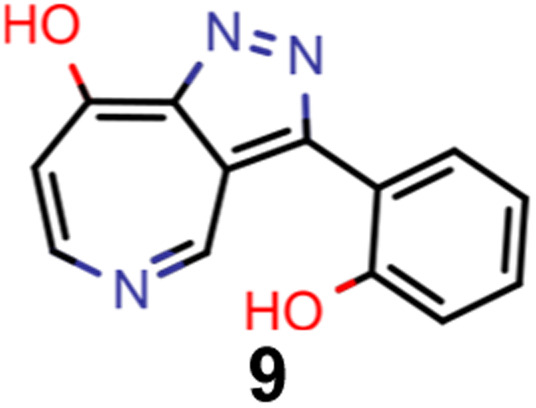	0.29	0.073	2.94
5	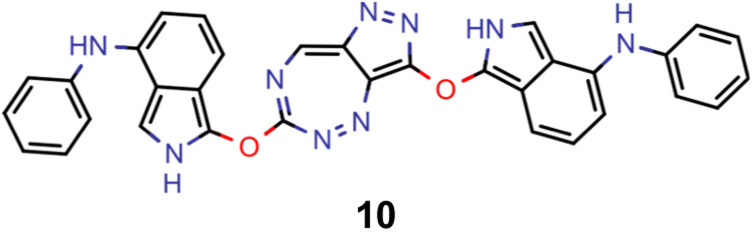	0.16	0.548	1.80
5	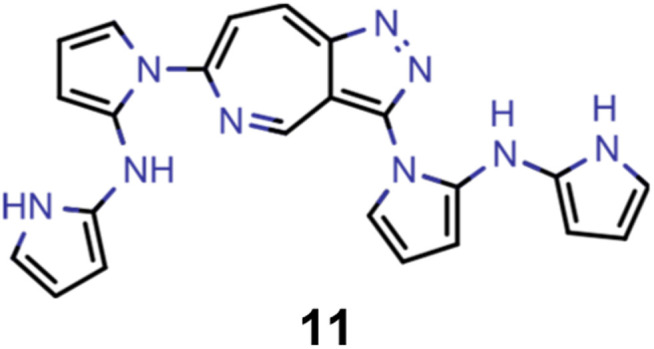	0.01	0.111	2.34
6	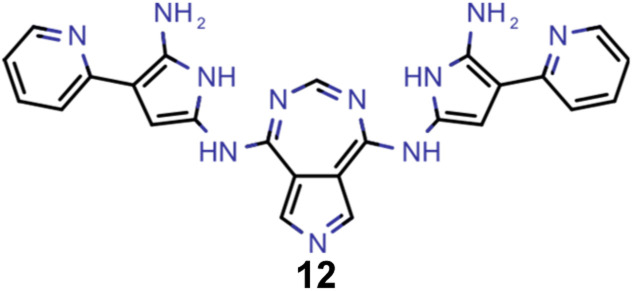	0.30	1.356	3.10
6	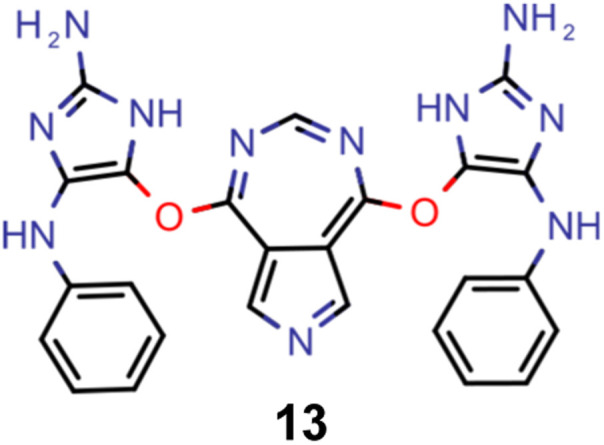	−0.07	0.529	2.54

Finally, we wanted to get insight into what the ANN classifiers, which were used as pre-filters for DFT simulations, learned in each of the experiments they were trained and used. To do that, we used the exmol package^60^ that implements the model agnostic counterfactual compounds with STONED (MACCS) methodology, that was recently developed. We adapted the corresponding workflow by implementing our filters for π-systems in the counterfactual generation to mimic the genetic operators of our GA. Additionally, while in the field of explainable artificial intelligence the generation of counterfactuals to understand decisions and predictions is well established,^61^ we were also interested in generating profactuals, *i.e.*, instances that are most similar to the reference and retain the same predictions. The idea is to not only find the smallest feature changes altering predictions^60,62^ but also to explore equally small feature changes not altering them. Accordingly, profactuals can be regarded as counterfactuals to the counterfactuals themselves and provide additional insight into the significance of counterfactual explanations.

Hence, we extended the implementation of MACCS to analyze both profactuals and counterfactuals in a consistent way. Subsequently, we applied this extended workflow to explain the predictions of the ANN classifiers based on the most promising candidates of each experiment listed in Table 2 except the first. The corresponding results for molecules 4–13 are illustrated in Supplementary Fig. 9–18.† Based on the structural comparison between the profactuals and counterfactuals, we find that changes to the core ring system are always counterfactuals. Additionally, the classifiers are sensitive to the nitrogen substitution pattern of the azulene π-system which is exemplified by some being regarded as acceptable and others being discarded. Furthermore, they are also sensitive to the type and position of substituents directly attached to the azulene core which is consistent with the bottom-up construction of INVEST molecules established recently. Moreover, some substituents, in particular when consisting of 4-membered and 8-membered ring systems, are always discarded regardless of whether they are directly attached to the core or further away. However, the classifiers are less sensitive to structural changes further away from the core ring system which is particularly apparent from the results for larger candidates where the introduction of additional substituents or the incorporation of heteroatoms is largely accepted. It should also be noted that substituent changes not affecting the electronic structure significantly are more likely to be accepted by the classifiers. Nevertheless, some counterfactuals correspond to structural changes that should not affect the properties of interest significantly. Similarly, some profactuals, in particular for the last two experiments with fixed substituent positions, break the corresponding constraints and, thus, move away from the structural space used for training.

### Lead validation

**Fig. 8:**
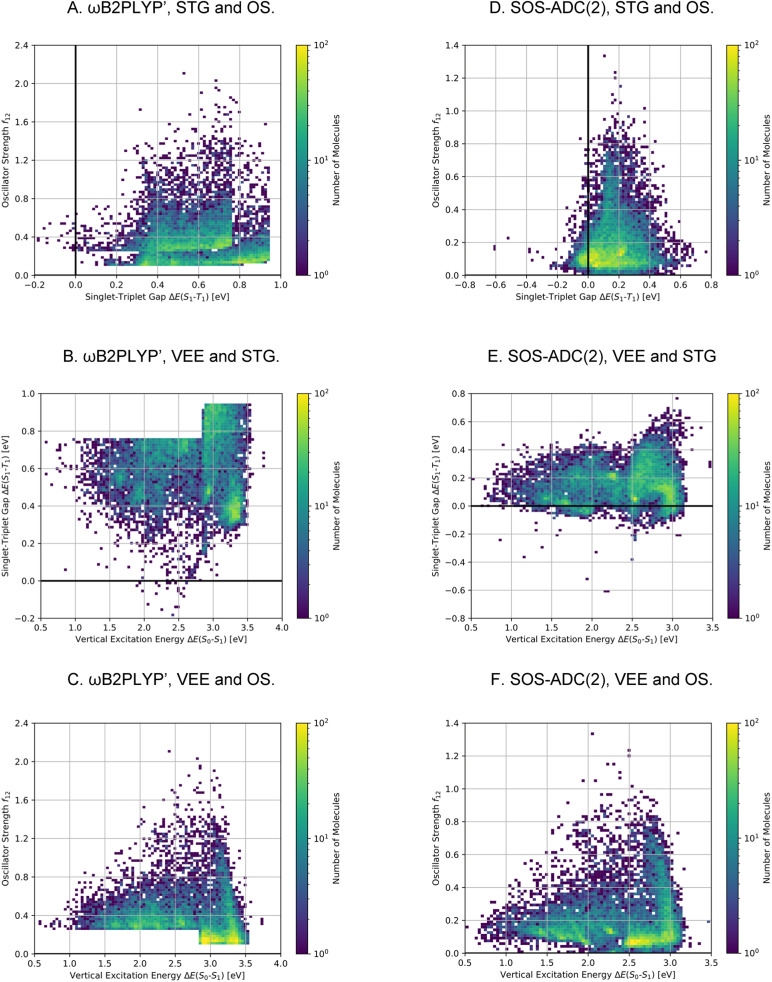
Property distributions of the validation set compounds at the ωB2PLYP’ (A)–(C) and the SOS-ADC(2) (D)–(F) levels of theory colored by the number of molecules in the respective property windows. STG: Singlet-triplet gap, OS: oscillator strength, VEE: vertical excitation energy.

After having found a large number of INVEST emitter candidates through artificial design, we proceeded to validate the best compounds across all runs using more reliable quantum chemistry simulations at the SOS-ADC(2) level of theory.^41,45–53^ This is important as the best-performing structures are significantly different from the initial candidates found in the high-throughput virtual screening. Additionally, using a different level of theory as employed by the genetic algorithm is key to check whether the algorithm exploited inherent methodology deficiencies. Accordingly, we combined the molecules from all experiments and applied Chimera^63^ to scalarize multiple objectives and select the best-performing molecules based on the resulting rankings. Thus, two independent rankings were established, one based on both STGs and OSs (Objective A), the other based on STGs, OSs and VEEs (objective B). In each of these rankings, the 7500 best molecules were selected for further validation, resulting in a total set of 13 222 unique compounds as some compounds appeared in both rankings. The corresponding property distributions at the ωB2PLYP’ and SOS-ADC(2) levels are depicted in Fig. 8 and the property correlations between the two methods are shown in Supplementary Fig. 19.† It should be noted that the distributions depicted in Fig. 8 result from concatenating two subsets with distinct property distributions. Consequently, the combined property distributions, especially at the ωB2PLYP’ level, show abrupt changes. These abrupt changes are much less pronounced at the SOS-ADC(2) level due to random noise when comparing the properties at the ωB2PLYP’ and SOS-ADC(2) levels (*cf.* Supplementary Fig. 19†).

Using SOS-ADC(2), 1310 (10%) of these compounds were predicted to have an inverted STG. Importantly, the relatively low number of confirmed INVEST molecules in the validation set mainly stems from the selection criteria and not from inaccuracies in the original predictions. We wanted to give the OS a considerable weight and focus on promising emitters rather than overemphasizing INVEST molecules with low oscillator strengths in the lead validation. This is evident from the ωB2PLYP’ properties of the validation compounds as only 1300 (10%) molecules have an STG below 0.36 eV. 566 of these 1300 compounds with lowest ωB2PLYP’ STGs are confirmed by SOS-ADC(2) to have an inverted STG, 1045 are predicted to have an STG lower than 0.10 eV based on SOS-ADC(2) results. This shows that ωB2PLYP’ simulations are not perfect predictors of STGs for the molecules investigated but they are sufficiently good in terms of accuracy to guide our artificial design workflow. Additionally, these results illustrate again the systematic offset between ωB2PLYP’ and SOS-ADC(2) (*cf.* Supplementary Fig. 1 and 19†). Using an STG of 0.36 eV at the ωB2PLYP’ level as heuristic to estimate the number of INVEST compounds in the entire set explored, we predict that there are 148 311 (17%) structures with inverted STG (*cf.*Table 1). The property distributions of this set of INVEST candidates are depicted in Fig. 7D–F. By requiring these INVEST candidates to have an OS of more than 0.05, there are in total likely 10 736 (1%) INVEST molecules with appreciable OS (*cf.*Table 1).

The property distributions at the SOS-ADC(2) level suggest that we successfully found organic molecules with both inverted STGs and OSs up to approximately 0.8 (*cf.*Fig. 8D). Additionally, we found INVEST molecules with VEEs spanning the entire visible light energy range (*cf.*Fig. 8E), and we also found emitters with appreciable OSs in that range (*cf.*Fig. 8F). Furthermore, the property correlations in the validation set indicate that while VEEs show excellent agreement between the two methods (*cf.* Supplementary Fig. 19A and B†), STGs and OSs of the validation set of high-performing candidates only show a moderate correlation between ωB2PLYP’ and SOS-ADC(2) (*cf.* Supplementary Fig. 19C–F†) indicating the optimization of these two properties in our workflow to be most challenging and that fine-tuning of STG and OS is difficult based on ωB2PLYP’ simulations.

A more cautious estimation of the number of potential INVEST molecules both in the validation set and in the full set of structures can be carried out by accounting for the systematic deviation between the EOM-CCSD, SOS-ADC(2), and ωB2PLYP’ levels observed in the nitrogen-substituted azulenes (*cf.*Fig. 2, Supplementary Fig. 1, and Table 1†). While it is not clear that this set of structures would show a similar systematic deviation between methods as the full set of structures generated by the genetic algorithm, especially because the underlying structures are not necessarily very similar, accounting for this deviation can still be insightful to provide a more careful estimate. When correcting all STG values at the SOS-ADC(2) level in the validation dataset, the number of candidates with inverted STGs is estimated to be reduced to 7. When doing the same with the values at the ωB2PLYP’ level, the number of candidates with inverted STG is estimated to be reduced to 923. This confirms that at least several INVEST candidates with appreciable OS were identified in the validation set, but it also suggests that the corresponding number is likely considerably smaller. Performing this type of correction for the entire set of structures using the ωB2PLYP’ results, the total number of candidates with inverted STGs is estimated to be 133 728 and the number of INVEST molecules with appreciable OS is estimated to be 8698, which is reasonably close to the estimates obtained *via* the alternative approach described above. Overall, these more cautious estimations show a considerable spread thus putting significant uncertainty on the number of INVEST candidates found. Nevertheless, all these additional estimates agree that many INVEST candidates were uncovered by our artificial design workflow.

We were also interested in the comparison of synthetic accessibility and complexity metrics between the entire set of compounds investigated, the structures predicted to possess an inverted STG, and a set of comparable reference structures that are known to be synthesizable. As no such dataset of reference structures existed, we created a subset of ZINC20 (ref. 64) containing 11 631 in-stock compounds that passed the filters used in the genetic algorithm (details in the supplementary computational details†). To quantify synthesizability, we used the synthetic accessibility score (SAscore),^65^ the synthetic complexity score (SCScore),^66^ the synthetic Bayesian accessibility metric (SYBA)^67^ and the retrosynthetic accessibility score (RAscore).^68^ In addition to providing an estimation as to how likely these molecules can be synthesized, at least some of them also incorporate an assessment of stability. First, we compared histograms of these metrics between the entire set of compounds generated during the artificial design stage, the subset of molecules estimated to possess an inverted STG, and the ZINC20 subset (*cf.* Supplementary Fig. 20†). They reveal that the subset of INVEST compounds does not have a considerably different distribution of synthesizability metrics. While the SAscore suggests them to be essentially identical, the SCScore indicates that the structural complexity is somewhat higher in the INVEST candidates. Compared to the ZINC20 subset, the SAscore distributions are considerably higher but there is still a significant overlap. The corresponding SCScore of the ZINC20 subset are also lower, but the overlap with compounds generated by the genetic algorithm is even larger. In contrast, using SYBA, the candidates are predicted to be somewhat more likely to be synthesizable. Similarly, the ZINC20 subset shows higher overlap of SYBA values to the algorithmically generated structures. The RAscore also shows the differences between all compounds generated and the subset of INVEST compounds not to be big. The corresponding differences are not only a consequence of the molecular properties themselves but also of the structural constraints employed in the artificial design experiments as demonstrated in Supplementary Fig. 21.† The runs with the largest fraction of INVEST compounds, *i.e.* experiments 5 and 6, have a large influence on the corresponding histograms. In contrast, experiment 1 largely only contributes to the histogram of all compounds as it has the lowest fraction of candidates estimated to have an inverted STG. Overall, while we find that these four metrics, based on their numeric values alone, suggest the majority of the compounds investigated to be likely synthesizable, the significant differences to the corresponding distributions of the ZINC20 subset suggest that synthesizability is not as high as readily available compounds. Notably, the corresponding threshold values for the SAscore has been suggested to be 4.5 and, for SYBA, −19.^67^ Additionally, the majority of compounds have an RAscore of 0.5 or higher, *i.e.*, it is very likely that AiZynthFinder^69^ will be able to propose a retrosynthetic route.

Finally, based on the properties at the SOS-ADC(2) level, six of the best candidates for each of the two objectives were selected. Their structures and simulated properties are provided in Table 3. Notably, all the compounds listed there emerged from experiment 6 and are likely stable. Additionally, they all possess at least two hydrogen-bond donors allowing for intramolecular interactions controlling their conformations. Importantly, for the tri-objective optimization of STG, OS and VEE, the target VEE for blue emitters at the SOS-ADC(2) level is 2.83 eV due to the systematic property differences relative to ωB2PLYP’ (*cf.* Supplementary Table 3†).

**Table 3:** Promising candidates after lead validation with their simulated properties at the SOS-ADC(2)/cc-pVDZ level of theory. Objective A refers to the optimization of singlet-triplet gap and oscillator strength, objective B refers to the optimization of singlet-triplet gap, oscillator strength and vertical excitation energy

Objective	Molecule	Δ*E*(S_1_–T_1_) [eV]	*f*_12_	Δ*E*(S_0_–S_1_) [eV]
A	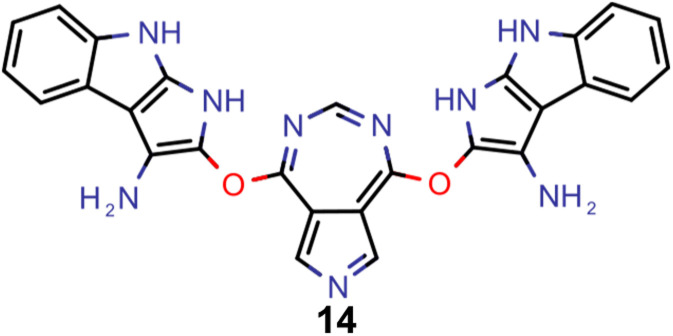	−0.01	0.401	2.26
A	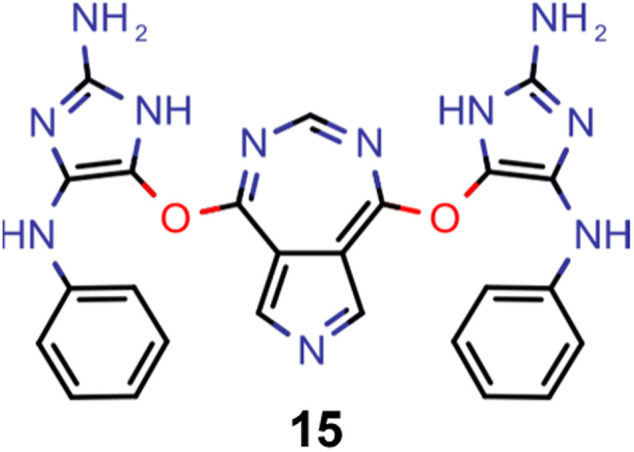	−0.01	0.336	2.19
A	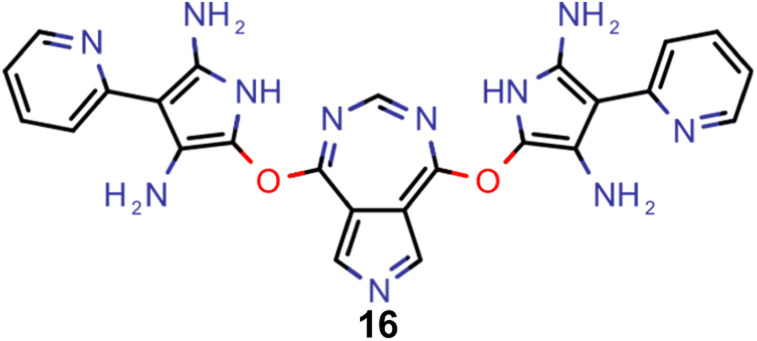	−0.02	0.298	2.38
A	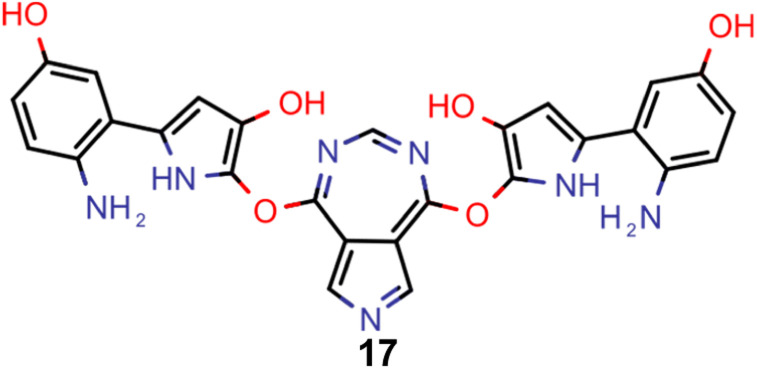	−0.39	0.137	2.51
A	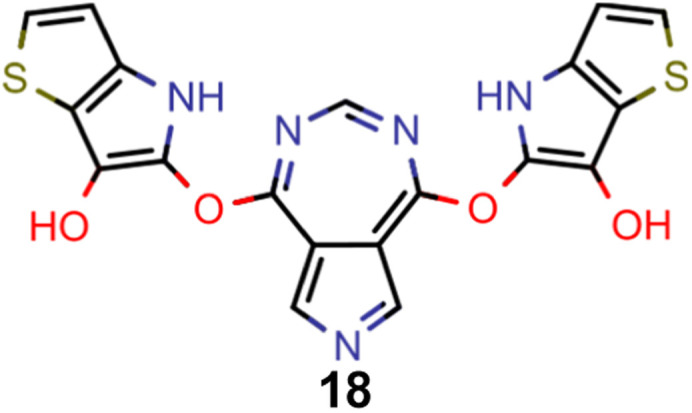	−0.11	0.169	2.50
A	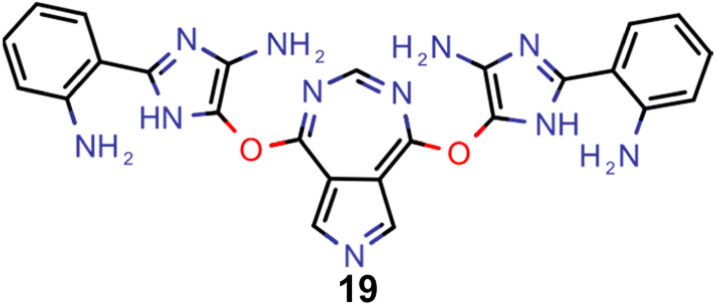	−0.08	0.268	2.38
B	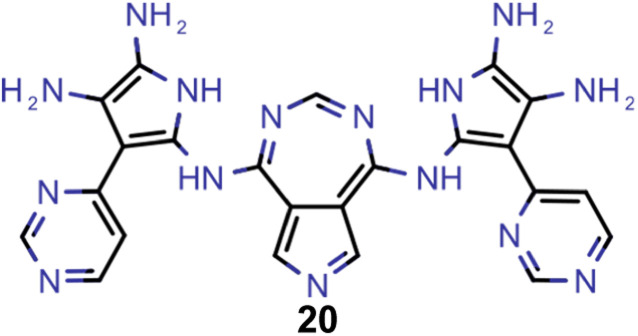	−0.02	0.307	2.79
B	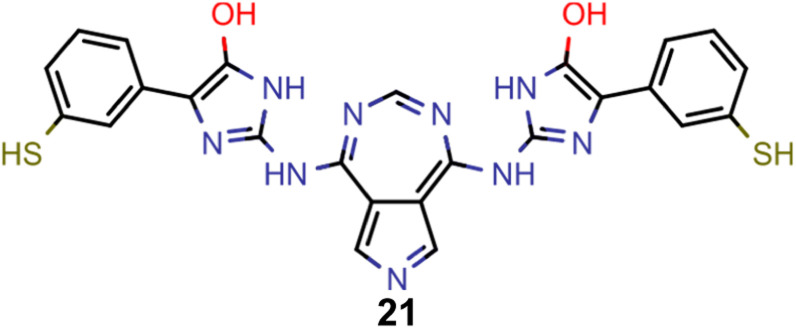	−0.01	0.305	2.86
B	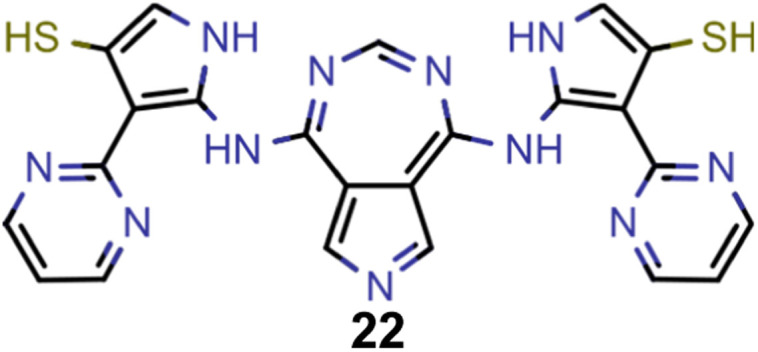	−0.03	0.296	2.83
B	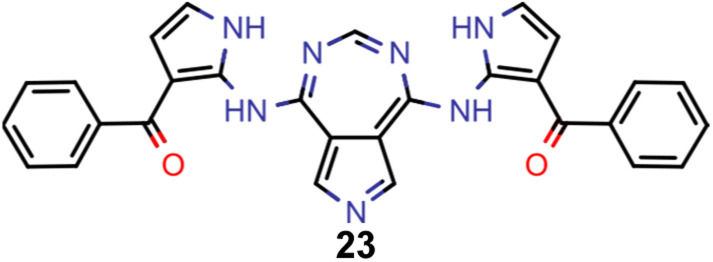	−0.11	0.121	2.79
B	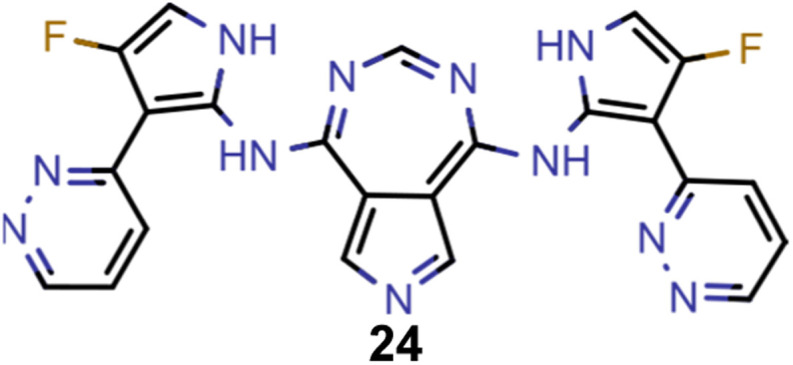	−0.10	0.132	2.79
B	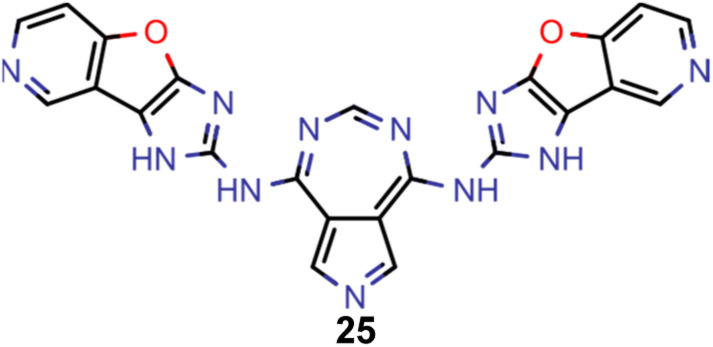	−0.08	0.111	2.84

## Discussion

We set out this work by establishing a comprehensive three-stage workflow for the artificial design of organic emitters relying on high-throughput virtual screening *via* quantum chemical simulations for property evaluation and a GA based on a robust molecular string representation enhanced by ANNs for efficient structure generation. After identifying promising core structures with inverted STGs *via* virtual screening, we explored the corresponding design space extensively, resulting in the generation of more than 800 000 emitter candidates with the goal to co-optimize STGs, OSs and VEEs. Overall, we found more than 10 000 candidates that likely possess both inverted STGs and appreciable OSs, many of which with predicted VEEs in the blue light energy range. In the following section, we will put our findings into perspective and outline future improvements for artificial design workflows.

In the first phase of our workflow, we developed and tested the simulation methodology, including the filters for π-systems, and defined the structural space to be explored. Our simulation protocol relies on both efficient and reliable methods to account for double excitations in the description of excited states, in particular double-hybrid time-dependent density functional approximations (DH-TD-DFAs),^37,70–73^ equation-of-motion coupled-cluster singles and doubles (EOM-CCSD),^54–58^ and second-order algebraic diagrammatic construction methods (ADC(2), SOS-ADC(2)),^38–53^ which is essential to describe molecules with inverted STGs appropriately.^21–23,74–76^ In the absence of reliable experimental reference data and robust experimental methods to characterize INVEST molecules, comparison against robust reference methods for the simulation of excited states such as ADC(2) and EOM-CCSD as performed in this work is a viable alternative to verify the validity of the simulated properties. Notably, even reliable methods such as EOM-CCSD have been shown to systematically overestimate STGs in related molecules,^29^ thus leading to more positive results. Thus, future research is required to verify our predictions experimentally.

Based on a combination of the INVEST design principles established previously^26^ and our virtual screening results, we selected azulenes as our core structures for further investigation. Importantly, while azulenes are notorious for violating Kasha's rule^77,78^ by emitting light mainly from their second rather than from their first excited singlet state,^79,80^ substituted azulenes emitting predominantly from their first excited electronic singlet states are known.^81^ As discussed previously,^26^ azulenes are promising candidates to realize INVEST emitters as fifth generation of organic light-emitting diode materials because they have intrinsically low STGs that can be inverted with proper modification, because they are stable structures with already several well-known synthetic pathways and because their excitation energies can be tuned over the entire visible light spectrum. To achieve that, the almost negligible OSs of the first electronically excited singlet states of azulene cores need to be enhanced with adequate structural substitution, which is why we chose azulenes as our target in this work. Future work will be necessary to understand the dominant excited state processes in substituted azulenes and enable conical intersection design in some of the most promising candidates.

Next, we set up our artificial design workflow by implementing the virtual screening approach into a development version of JANUS,^11^ a GA relying on SELFIES^1^ as representation and the STONED algorithm^2^ for robust and efficient molecular structure generation. One of the advantages of this approach is that it can be applied to any molecular design problem with a well-defined fitness function without prior knowledge of the structural space to be investigated. Additionally, it allows us to incorporate domain knowledge, which is what we did by enforcing our filters for π-systems in the molecular generation. These filters are the main reason that, in experiment 1, with methane as seed, azulene was rediscovered already in the first generation. We rationalize this observation by azulene being a very simple π-system with only two annulated rings satisfying our filters. To the best of our knowledge, it is one of the simplest core structures promoting inverted STGs.^26^ The filters were designed to avoid the exploration of structures that are unlikely to lead to sizable improvement of the properties we simulated but likely to distract our artificial design workflow and make the property simulations more time-consuming. Notably, while alkyl groups can lead to favorable device properties in organic light-emitting diodes, the corresponding impact is not captured by the simulated excited state properties and fitness functions we employed. Accordingly, we did not allow for the presence of alkyl groups in any of the molecules generated. Importantly, we believe this to be one of the reasons for the high number of hydrogen-bonding moieties in many of the best-performing molecules found. Our workflow allows for amines, alcohols and thiols to be introduced as electron-donating groups but cannot satisfy the corresponding valences with alkyl groups that would also make them more stable. The potential benefit of introducing alkyl groups into the best candidates we found is beyond the scope of this work as it requires to extend the range of properties considered and needs to be addressed in future studies.

Importantly, while we applied the artificial molecular design workflow to find potential blue emitters, the workflow we developed is not limited to this particular excitation energy range. It is general and can be applied to any excitation energy range of interest such as green, red, or even near infrared emitters. This can simply be done by changing the fitness function of the genetic algorithm, specifically the term that incorporates the excitation energy. By choosing a different target value for the excitation energy, emitters with different colors can be designed.

Furthermore, we found it to be crucial to narrow down the design space continuously as we explored more structures. This is demonstrated by experiments 5 and 6 where we constrained the substituent positions in the azulene cores and required the substituents to be identical. This led to a dramatic improvement of the inverse design and molecules with superior properties. We believe that this inability to narrow down the space to be investigated autonomously is still one weakness of the JANUS version we employed in this work. It has been partially addressed already in the published version of JANUS,^11^ and we aim to improve upon this issue in upcoming work even further. Moreover, using the generation of both counterfactuals and profactuals, we obtained insight into what the ANN classifiers learned. In that regard, it is encouraging to observe that changes to the core structure are regarded as crucial whereas modifications further away are more readily accepted which is essential to enhance the sampling of promising candidates.

Finally, in the lead validation stage, we confirmed the findings of the artificial design by performing more reliable quantum chemistry simulations of the excited state properties. The method we adopted for that purpose, SOS-ADC(2), is considered one of the state-of-the-art approaches to simulate excited state properties for molecules of considerable size, especially INVEST compounds. Altogether, we identified more than 1000 candidates for INVEST emitters with appreciable OS in the validation set, and estimate that there are more than 10 000 in the full set of compounds explored in this work. Notably, this is more than one order of magnitude larger than the number of INVEST emitters found in out previous high-throughput virtual screening approach relying on expert design.^21^ This vast number of molecules with both inverted STGs and considerable OSs shows that the INVEST compound space is much larger than initially thought,^22,23^ and that artificial molecular design enables the comprehensive exploration of extreme property spaces with unprecedented efficiency.

Ultimately, the findings in this work need to be verified in the laboratory. While many of the molecules proposed are likely stable, due to the intrinsic stability of azulenes, most of the azaazulene core structures explored have never been synthesized. In particular, to the best of our knowledge, 2,5,7-triazaazulene (molecule 1) has not been reported before. This suggests that the results of the synthetic accessibility and complexity metrics should be interpreted with care. They likely indicate that there is no obvious structural feature that makes the proposed compounds hard to synthesize. However, the lack of literature precedence suggests that the metrics are applied outside their original application domain and, hence, cannot be expected to give a highly reliable estimation of whether these compounds can actually be synthesized. In particular, fully conjugated nitrogen-containing heterocycles are generally not straightforward to synthesize as they often require distinct synthesis routes. This demonstrates that new synthetic approaches for these compounds need to be developed before azulene-based INVEST emitters can unlock their full potential as organic electronic materials. Accordingly, we hope that our findings will inspire other groups to explore the synthesis of azaazulenes and their substituted derivatives, and realize some of the most promising emitter candidates that were proposed in our workflow. Overall, our work showcases the combination of state-of-the-art quantum chemistry simulations and artificial molecular design infused with machine learning and domain knowledge to tackle real-world design challenges in chemistry. Accordingly, we believe that the inverse molecular design workflow implemented in this work can serve as a model for future studies defining a new standard for accelerated inverse design campaigns.

## Computational methods

### High-throughput virtual screening

Ground state conformational ensembles were generated using crest^82^ (version 2.10.1) with the iMTD-GC^83,84^ workflow (default option) using the GFN2-xTB^85,86^//GFN-FF^87–89^ composite method. The composite method was selected as it provides comparable results to the use of GFN2-xTB for the full workflow at a fraction of the computational cost. The lowest energy conformers were first reoptimized using xtb^90^ (version 6.3.0) at the GFN2-xTB^85,86^ level of theory, to reduce the number of required subsequent optimization steps, followed by another reoptimization using Orca^91,92^ (version 4.2.1) at the B97-3c^93^ level of theory, which has been shown to be a good choice for accurate ground-state geometry optimizations at comparably low computational cost. Notably, accurate ground-state structures are a prerequisite for reliable vertical excited state properties. The corresponding geometries were used for subsequent ground and excited state single-point calculations. Single points at the RKS-ωB2PLYP’^37^/def2-mSVP^94^ level of theory were performed using Orca^91,92^ (version 4.2.1), single points at the RI-ADC(2)^38–44^/cc-pVDZ^95^ and the RI-EOM-CCSD^54–58^/cc-pVDZ^95^ levels of theory were performed using Q-Chem^96^ (version 5.2). Single points at the RI-SOS-ADC(2)^41,45–53^/cc-pVDZ^95^ level of theory were performed using MRCC^97^ (version 2020). Importantly, in the Orca version used (version 4.2.1), the perturbative doubles correction is not applied to the excited triplet states when using restricted Kohn–Sham (RKS) calculations.^98^ Hence, to indicate this explicitly in our results, we term the corresponding method ωB2PLYP’ as opposed to ωB2PLYP. Simulations at the RI-SOS-ADC(2) level of theory were performed with 9 roots in the singlet and 8 roots in the triplet manifold. Hence, 8 excited roots were selected for both. For all other excited state single point calculations, four roots were chosen each for both the singlet and the triplet manifold. The filters used to create the π-systems subset of GDB-13 (ref. 36) were implemented using RDKit^99^ and are summarized in Table 4. The source code of these filters can be found in our GitHub repository ¶¶GitHub: https://github.com/aspuru-guzik-group/Artificial-Design-of-Organic-Emitters..

**Table 4:** List of filters employed to create the π-systems subset of GDB-13

Number	Feature	Definition	Value
1	Charge	Charge of the molecule	*x* = 0
2	Radicals	Number of radical electrons	*x* = 0
3	Bridgehead atoms	Number of bridgehead atoms	*x* = 0
4	Spiro atoms	Number of spiro atoms	*x* = 0
5	Aromaticity degree	Percentage of aromatic non-hydrogen atoms	*x* ≥ 0.5
6	Conjugation degree	Percentage of conjugated bonds between non-hydrogen atoms	*x* ≥ 0.7
7	Maximum ring size	Size of the largest ring	4 ≤ *x* ≤ 8
8	Minimum ring size	Size of the smallest ring	4 ≤ *x* ≤ 8
9	Substructures	List of forbidden substructures. The code can be found in the GitHub repository	False

### Artificial design

Simulations of excited state properties for fitness evaluation were carried out as described in the previous section by generation of conformational ensembles using crest,^82^ geometry optimizations at the GFN2-xTB^85,86^ and the B97-3c^93^ levels of theory and single points at the RKS-ωB2PLYP’^37^/def2-mSVP^94^ level of theory.

Artificial design was performed using a development version of JANUS,^11^ a genetic algorithm (GA) for molecular design. Every run was seeded with a single molecule (*cf.*Table 5). The first generation in each run was created from random mutations of the seed using the STONED algorithm.^2^ All genetic operations with STONED were performed using version 1.0.1 of SELFIES.^1^ The fitness of each molecule was evaluated as a sum of three fitness components (*cf.*Table 5), one for each property of interest, namely, singlet-triplet gap (STG, Δ*E*(S_1_–T_1_)), oscillator strength (OS, *f*_12_) and vertical excitation energy (VEE, Δ*E*(S_0_–S_1_)). In case any of the properties of interest carries a unit, we formally divide the corresponding property by a property value of unity with the same unit, which leads to dimensionless numbers for all properties. These dimensionless numbers were then used for arbitrary linear combinations. Additionally, for each of the fitness components, very low fitness values of −10^6^ were assigned when the properties did not fulfill minimum requirements. For the STG component, the corresponding fitness value was required to be non-negative. For the OS component, the corresponding fitness value was required to be non-negative. For the VEE component, the property value was required to be non-negative. The molecules in each generation were ranked based on the fitness from best, *i.e.*, highest fitness value, to worst, *i.e.*, lowest fitness value. The top 20% of each generation were propagated to the subsequent one. The other molecules were replaced by structures generated by the genetic operators applied to the top 20%. The molecules in each generation were required to be unique across all previous generations during each experiment, which was checked explicitly in the genetic operators by maintaining a dictionary of all previous structures. The number of atoms in each molecule was capped at 70 throughout this work. Additionally, the filters developed in the virtual screening were used in the genetic operators to only generate structures satisfying them. The source code of these filters can be found in our GitHub repository. The number of molecules per generation was capped at 10 000. All experiments except for the first were stopped after generation 15, experiment 1 was stopped after generation 11 (*cf.*Table 5).

**Table 5:** Setup details of the genetic algorithm with respect to seed molecule, fitness function and the number of generations for each artificial design run. STG: singlet-triplet gap, OS: oscillator strength, VEE: vertical excitation energy

Run	Seed molecule	STG fitness	OS fitness	VEE fitness	Generations
1	Methane	0.6 − Δ*E*(S_1_–T_1_)	*f*_12_	0	11
2	2,5,7-Triazaazulene	0.3 − Δ*E*(S_1_–T_1_)	*f*_12_	0	15
3	2,5,7-Triazaazulene	0	*f*_12_	0	15
4	2,5,7-Triazaazulene	0.3 − Δ*E*(S_1_–T_1_)	*f*_12_	−|Δ*E*(S_0_–S_1_) − 3.2|	15
5	2,5,7-Triazaazulene	0.3 − Δ*E*(S_1_–T_1_)	*f*_12_	0	15
6	2,5,7-Triazaazulene	0.3 − Δ*E*(S_1_–T_1_)	*f*_12_	0	15

Subsequently, for all runs except for the first, an artificial neural network (ANN) classifier was incorporated into the GA after generation 11. For each experiment, the data from the first 11 generations were collected and used to train a fully-connected 2-layer ANN classifying molecules as either good (*i.e.*, output of 1) or bad (*i.e.*, output of 0). As molecular features, we used the binary representation of Morgan fingerprints^100^ consisting of 1024 bits. In the data from previous generations, all structures with an STG below 0.6 and an OS larger than 0.0 were classified as good, the others as bad. These data were split into three separate sets. First, 20% of the data were used as a holdout set to test model performance. The remaining 80% was split again into 48% of the total used for training and 32% of the total used as validation set. The validation set was used to tune hyperparameters with the package Optuna.^101^ In that regard, we decided to optimize the number of training epochs, the number of epochs to continue training without validation loss improvement, the learning rate, the number of neurons in each layer and the dropout rate. The final classification accuracy of the models was evaluated based on the holdout set (*cf.*Table 1). Classification accuracy was calculated as the percentage of molecules that was classified correctly as either good or bad. Subsequently, the classifiers were incorporated into the genetic operators of each run and combined with the other filters used therein (*vide supra*). Only molecules classified as good were passed on to the fitness evaluation *via* property simulation, molecules classified as bad were discarded. Our choice to incorporate a classifier was influenced by an early attempt to use ANNs predictors of singlet-triplet gaps and oscillator strengths. However, we found direct property prediction to be hard and only obtained poor correlations (Supplementary Table 2†).

Finally, to get insight into what the ANN classifiers learned, we used the exmol package (version 0.6.0).^102^ We modified the default workflow established in that package by implementing the filters we developed in the virtual screening to only generate structures satisfying them as potential counterfactuals. Additionally, we also added the generation of profactuals, *i.e.*, molecules in the structural vicinity of the reference that still retains the same classification, to the workflow. For each baseline molecule, 9 profactuals and 9 counterfactuals were generated. Sampling was performed *via* the STONED algorithm with version 1.0.4 of SELFIES^1^ using the medium settings implemented in exmol but increasing the number of samples to 15 000. The corresponding source code can be found in our GitHub repository.

### Lead validation

The best candidates generated throughout all the artificial design experiments were selected using Chimera.^63^ Two separate rankings were performed, one based on a bi-objective optimization of both STGs and OSs, another based on a tri-objective optimization of STGs, OSs and VEEs. The corresponding parameters used in Chimera for these two rankings are provided in Table 6. The 7500 best candidates in each of these two rankings were concatenated and the corresponding molecules were validated with a more reliable computational method. To validate the properties of the selected candidates, the geometries at the B97-3c^93^ level of theory obtained from the fitness evaluation were used for subsequent single point calculations at the RI-SOS-ADC(2)^41,45–53^/cc-pVDZ^95^ level of theory.

**Table 6:** Chimera parameters to perform (A) bi-objective optimization of singlet-triplet gap and oscillator strength and (B) tri-objective optimization of singlet-triplet gap, oscillator strength and excitation energy

Objectives	Tolerances	Absolutes	Goals
(A) Bi-objective optimization			
(1) Singlet-triplet gap	5.00	True	Minimize
(2) Oscillator strength	0.35	True	Maximize

(B) Tri-objective optimization			
(1) Singlet-triplet gap	3.000	True	Minimize
(2) Oscillator strength	0.175	True	Maximize
(3) Absolute difference of excitation energy to 3.2 eV	0.350	True	Minimize

## Data availability

Detailed results are provided in our GitHub repository: https://github.com/aspuru-guzik-group/Artificial-Design-of-Organic-Emitters.

## Code availability

Code to run our experiments are provided in our GitHub repository: https://github.com/aspuru-guzik-group/Artificial-Design-of-Organic-Emitters.

## Author contributions

A. N. and R. P. conceived the idea of the project. P. F. and R. P. designed the simulation methodology, performed the high-throughput virtual screening and analyzed the corresponding results. A. N. developed the code of the genetic algorithm. A. N. and R. P. designed the genetic algorithm setup, performed the corresponding computations and analyzed the respective results. All authors discussed and refined the scientific results. The manuscript was mainly written by A. N. and R. P. with input from all other authors.

## Conflicts of interest

The University of Toronto has filed a provisional application for a US patent based on the technology described in this paper, naming A. N., R. P., P. F., and A. A.-G. as inventors. A. A.-G. is co-founder and Chief Visionary Officer of Kebotix, Inc.

## Supplementary Material

SC-015-D3SC05306G-s001
