# Supplementary material for: Artificial design of organic emitters via a genetic algorithm enhanced by a deep neural network
Source: Chem Sci. 2024 Jan 11;15(7):2618–39. doi: 10.1039/d3sc05306g (PMC10866360; doi:10.1039/d3sc05306g)
Supplement: SC-015-D3SC05306G-s001 [file SC-015-D3SC05306G-s001.pdf]

Electronic Supplementary Material (ESI) for Chemical Science.
This journal is © The Royal Society of Chemistry 2024

# Supplementary Information

# Artificial Design of Organic Emitters via a Genetic Algorithm Enhanced by a Deep Neural Network

AkshatKumar Nigam,[1,2,11] Robert Pollice,*,[1,2,3,11] Pascal Friederich[1,2,4,5] and Alán Aspuru-Guzik*,[1,2,6,7,8,9,10]

[1] Chemical Physics Theory Group, Department of Chemistry, University of Toronto, 80 St. George St, Toronto, Ontario M5S 3H6, Canada.

[2] Department of Computer Science, University of Toronto, 40 St. George St, Toronto, Ontario M5S 2E4, Canada.

[3] current affiliation: Stratingh Institute for Chemistry, University of Groningen, Nijenborgh 4, Groningen, 9747 AG, The Netherlands.

[4] Institute of Nanotechnology, Karlsruhe Institute of Technology, Hermann-von-Helmholtz-Platz 1, 76344 Eggenstein-Leopoldshafen, Germany.

[5] Institute of Theoretical Informatics, Karlsruhe Institute of Technology, Am Fasanengarten 5, 76131 Karlsruhe, Germany.

[6] Vector Institute for Artificial Intelligence, 661 University Ave Suite 710, Toronto, Ontario M5G 1M1, Canada.

[7] Department of Chemical Engineering & Applied Chemistry, University of Toronto, 200 College St., Ontario M5S 3E5, Canada.

[8] Department of Materials Science & Engineering, University of Toronto, 184 College St., Ontario M5S 3E4, Canada.

[9] Lebovic Fellow, Canadian Institute for Advanced Research (CIFAR), 661 University Ave, Toronto, Ontario M5G, Canada.

[10] Acceleration Consortium, Toronto, Ontario M5G 3H6, Canada.

[11] These authors contributed equally.

*Corresponding authors: r.pollice@rug.nl, aspuru@utoronto.ca*

GitHub: https://github.com/aspuru-guzik-group/Artificial-Design-of-Organic-Emitters.

## Supplementary Computational Methods

**Virtual Screening.** All systematic permutations of nitrogen substitutions of azulene were generated with the itertools package of python via the itertools.product function to generate all permutations of sequences of the characters 'C' and 'N' of length 8. These sequences were then embedded into the azulene skeleton by placing the corresponding characters at the 8 distinct positions of the SMILES string that allow for nitrogen substitution. The resulting SMILES were canonicalized via the rdkit package. Duplicate SMILES in the resulting list were removed by creating a set. The code of this procedure can be found on the GitHub repository.

**Lead Validation.** As a reference dataset for comparing the synthesizability scores of the structures generated by our algorithm to, we downloaded the subset of ZINC20[1] that contains all the compounds in stock (In-Stock tag, https://zinc.docking.org/tranches/home/). It encompasses over 13 million compounds. To reduce computational expense, we sampled a random subset containing 20% of this full set. We filtered the resulting subset with the same

filters that used to filter GDB-13, that we also employed in our genetic algorithm. This yielded 11,631 structures, for which we computed the SAscore, the SCScore, SYBA, and the RAscore.

## Supplementary Results and Discussion

**Virtual Screening.** The comparison of simulated properties at the EOM-CCSD/cc-pVDZ level of theory against alternative methods is illustrated in Supplementary Figure 1. The results show that all three methods tested, i.e., ωB2PLYP'/def2-mSVP, ADC(2)/cc-pVDZ and SOS-ADC(2)/cc-pVDZ reproduce the properties and the corresponding trends well (cf. Supplementary Table 1). Overall, SOS-ADC(2)/cc-pVDZ shows the best agreement across singlet-triplet gaps, oscillator strengths and vertical excitation energies, as demonstrated by the results presented in Supplementary Table 1. Accordingly, we decided to employ ωB2PLYP'/def2-mSVP for property simulation in our artificial design stage as it is computationally the most efficient method. Additionally, we selected SOS-ADC(2)/cc-pVDZ as our method of choice to validate some of the best candidates that were found in the artificial design stage. ADC(2)/cc-pVDZ shows somewhat worse agreement with EOM-CCSD/cc-pVDZ at a higher computational expense.

**Supplementary Table 1.** Linear regression results of simulated excited state properties for all systematic permutations of nitrogen core structure substitutions of azulene at the EOM-CCSD level of theory against the corresponding properties obtained from alternative methods: Y = Slope · X + Intercept. $R^2$ corresponds to the coefficient of determination. F corresponds to the result of an f-test that compares the model to parameter values of 0. N corresponds to the number of data points. N is lower than 144 for SOS-ADC(2) as 4 simulations did not converge.

| Method | Property | Slope | Intercept | $R^2$ | F | N |
|---|---|---|---|---|---|---|
| ADC(2) | $\Delta E(S_0-S_1)$ | 0.90(4) | -0.05(10) | 0.79 | 521 | 144 |
| ADC(2) | $\Delta E(S_1-T_1)$ | 0.56(3) | -0.006(15) | 0.72 | 366 | 144 |
| ADC(2) | $f_{12}$ | 0.80(6) | 0.003(10) | 0.59 | 201 | 144 |
| SOS-ADC(2) | $\Delta E(S_0-S_1)$ | 0.97(2) | -0.04(5) | 0.95 | 2419 | 140 |
| SOS-ADC(2) | $\Delta E(S_1-T_1)$ | 1.08(4) | -0.30(2) | 0.87 | 924 | 140 |
| SOS-ADC(2) | $f_{12}$ | 0.73(3) | 0.001(0) | 0.83 | 667 | 140 |
| ωB2PLYP' | $\Delta E(S_0-S_1)$ | 0.93(3) | 0.28(7) | 0.90 | 1246 | 144 |
| ωB2PLYP' | $\Delta E(S_1-T_1)$ | 0.79(5) | 0.34(3) | 0.62 | 236 | 144 |
| ωB2PLYP' | $f_{12}$ | 1.26(8) | 0.003(1) | 0.65 | 259 | 144 |

A. Comparison of Vertical Excitation Energies

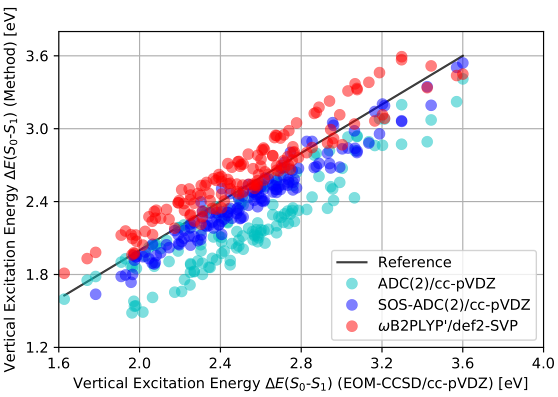


B. Comparison of Singlet-Triplet Gaps

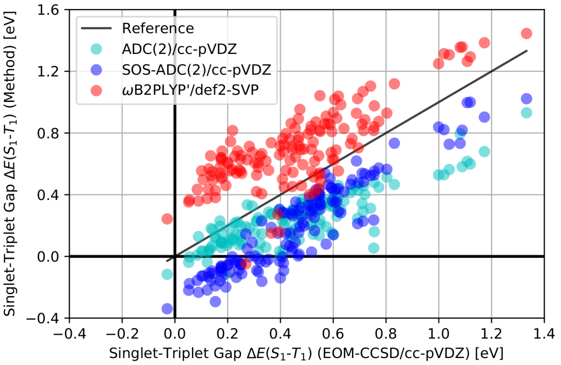


C. Comparison of Oscillator Strengths

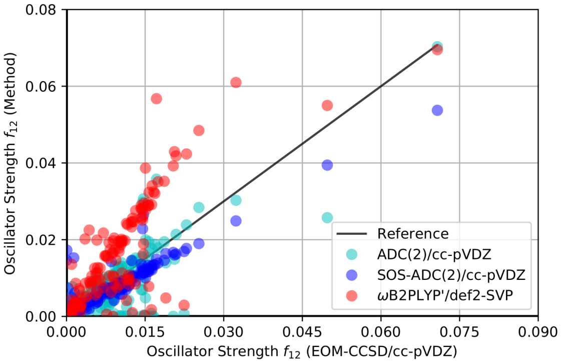


**Supplementary Figure 1.** Comparison of simulated excited state properties for all systematic permutations of nitrogen core structure substitutions of azulene at the EOM-CCSD level of theory against the corresponding properties obtained from alternative methods. A. Comparison of vertical excitation energies. B. Comparison of singlet-triplet gaps. C. Comparison of oscillator strengths.

**Artificial Design.** As it is relevant throughout this work, especially for our artificial design experiments, Supplementary Figure 2 shows the numbering of positions in azulene core structures. This numbering is used to refer to both the positions of nitrogen substitutions in the core structure and the introduction of additional substituents.

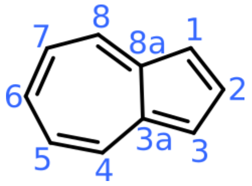


**Supplementary Figure 2.** Numbering of positions in azulene.

In the following paragraph, we will describe our initial attempts to train two ANN property predictors, one for singlet-triplet gaps and one for oscillator strengths. First, 20% of the data were used as a holdout set to test model performance. The remaining 80% were split again into 48% of the total used for training and 32% of the total used as validation set. The validation set was used to tune hyperparameters with the package Optuna.[2] In that regard, we decided to optimize the number of epochs for training, the number of epochs to continue training without validation loss improvement, the learning rate, the number of neurons in each layer and the dropout rate. We trained several ANNs for each of the two properties from the data generated in the first 11 generations of the artificial design experiment 1 using various input features for comparison as detailed in Supplementary Table 2. Each row corresponds to a different set of features used for training. Depending on the features, the ANNs consisted of either two dense layers, i.e., fully connected neurons (cf. SELFIES, SMILES, ECFP4 and ECFP4-XTB in Supplementary Table 2), or two layers of graph convolution operations (cf. GCN in Supplementary Table 2) as implemented in GCNConv of the deep graph library.[3] The SELFIES and SMILES models used one-hot encodings of the respective SELFIES and SMILES of the molecules. The ECFP4 models used the binary representation of Morgan fingerprints[4] with 1024 bits. In addition to the Morgan fingerprints, the ECFP4-XTB models also used various molecular properties derived from semiempirical quantum chemistry simulations of the molecules. These simulations were initiated by ground state conformational ensembles via crest[5] (version 2.10.1) with the iMTD-GC[6,7] workflow (quick option) using the GFN-FF[8–10] method in conjunction with loose optimization settings. Subsequently, the lowest energy conformers were optimized using xtb[11] (version 6.3.0) at the GFN2-xTB[12,13] level of theory using normal geometry convergence. From the output of the GFN2-xTB simulations, we extracted the following molecular properties as features: the three molecular rotational constants, the three molecular moments of inertia, the molecular dipole moment vector and the corresponding

norm, the six molecular quadrupole moments, the homoatomic $C_6^{AA}$ and $C_8^{AA}$ dispersion coefficients, the molecular polarizability, the fermi energy level, the energy difference between the highest occupied molecular orbital and the lowest unoccupied molecular orbital, the energies of the six highest occupied molecular orbitals and the energies of the six lowest unoccupied molecular orbitals. For the GCN models, we reimplemented the code and features used in Chemprop[14] to train graph convolutional neural networks. As apparent from Supplementary Table 2, none of the models show sufficiently good prediction performance compared to the quantum chemical simulations, neither for singlet-triplet gaps nor for oscillator strengths. Hence, we decided to incorporate ANN classifiers into the artificial design workflow instead.

**Supplementary Table 2.** Holdout set performance for predicting singlet-triplet gaps ($\Delta E(S_1\text{-}T_1)$) and oscillator strengths ($f_{12}$). MAE: mean absolute error, RMSE: root mean squared error, $R^2$: coefficient of determination.

| Model Features | $\Delta E(S_1\text{-}T_1)$ [eV] | | | $f_{12}$ | | |
|---|---|---|---|---|---|---|
| | MAE | RMSE | $R^2$ | MAE | RMSE | $R^2$ |
| SELFIES | 0.25 | 0.33 | 0.375 | 0.08 | 0.19 | 0.239 |
| SMILES | 0.24 | 0.32 | 0.413 | 0.08 | 0.19 | 0.282 |
| ECFP4 | 0.20 | 0.27 | 0.580 | 0.07 | 0.16 | 0.435 |
| GCN | 0.20 | 0.26 | 0.625 | 0.09 | 0.18 | 0.322 |
| ECFP4-XTB | 0.18 | 0.24 | 0.669 | 0.06 | 0.15 | 0.547 |

In addition to the property distributions provided in the Extended Data section of the main text, we also provide property distributions at the ωB2PLYP' level of theory for all structures generated in each of the artificial design runs (cf. Supplementary Figures 3-8). They illustrate several relationships between the properties of interest. First, as is already well established, we find that molecules with smaller singlet-triplet gaps tend to have smaller oscillator strengths. Additionally, molecules with smaller singlet-triplet gaps also tend to have smaller vertical excitation energies, and we also find that structures with predicted vertical excitation energies around 3 eV tend to have the largest oscillator strengths.

A. Singlet-triplet gap and oscillator strength.

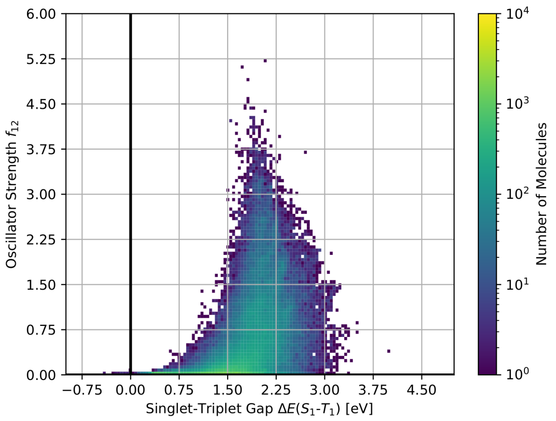


B. Vertical excitation energy and singlet-triplet gap.

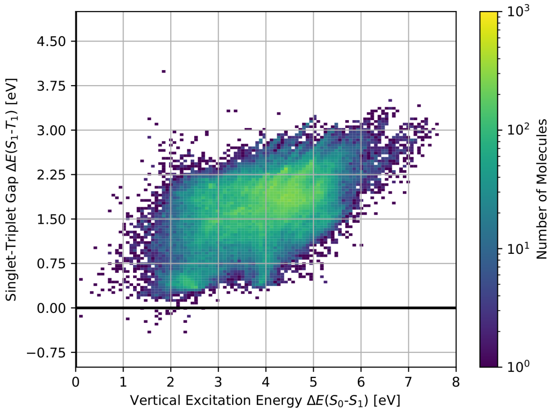


C. Vertical excitation energy and oscillator strength.

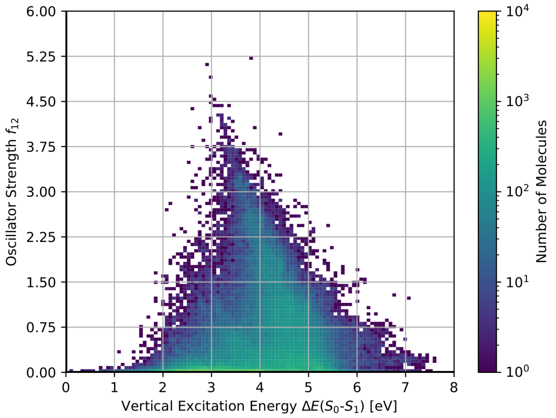


**Supplementary Figure 3.** Property distributions of all the compounds generated during experiment 1 of the artificial design stage at the ωB2PLYP' level of theory colored by the number of molecules in the respective property windows (A-C).

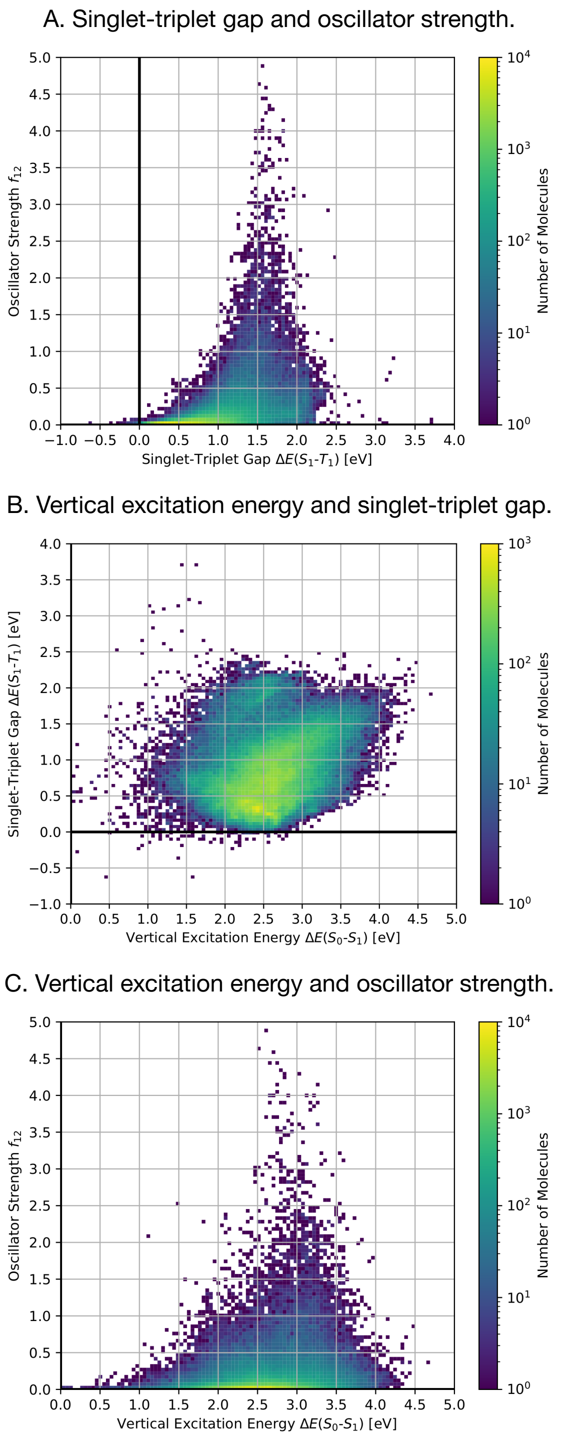


**Supplementary Figure 4.** Property distributions of all the compounds generated during experiment 2 of the artificial design stage at the ωB2PLYP' level of theory colored by the number of molecules in the respective property windows (A-C).

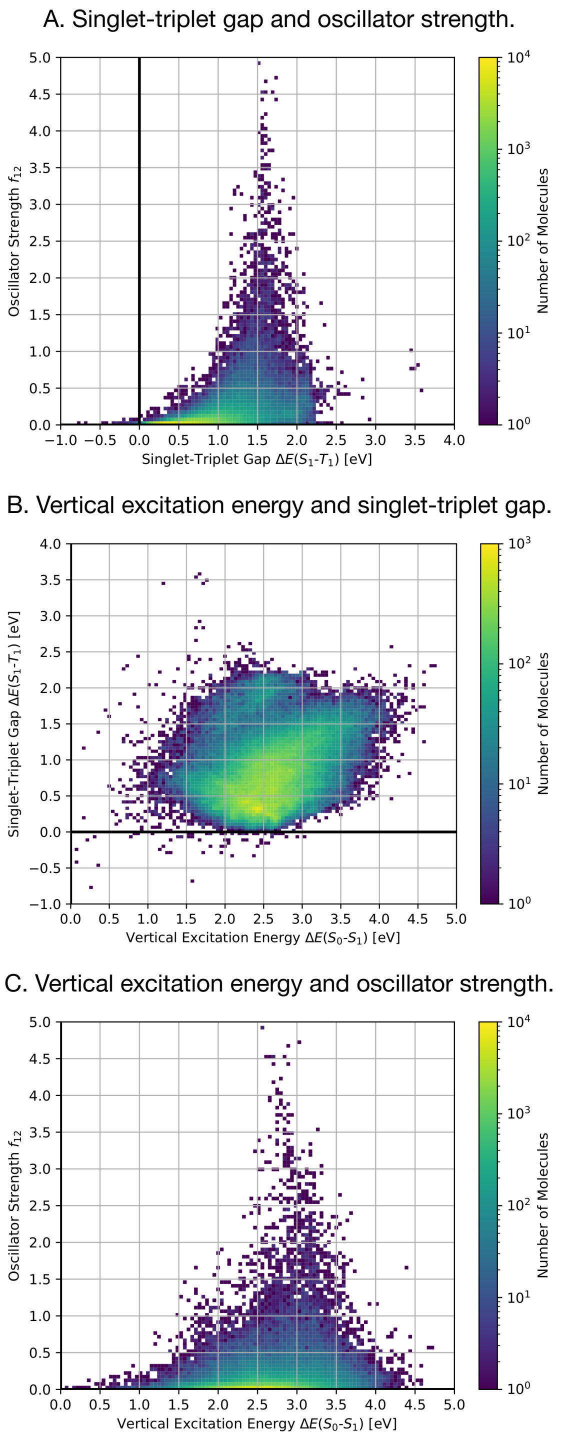


**Supplementary Figure 5.** Property distributions of all the compounds generated during experiment 3 of the artificial design stage at the ωB2PLYP' level of theory colored by the number of molecules in the respective property windows (A-C).

A. Singlet-triplet gap and oscillator strength.

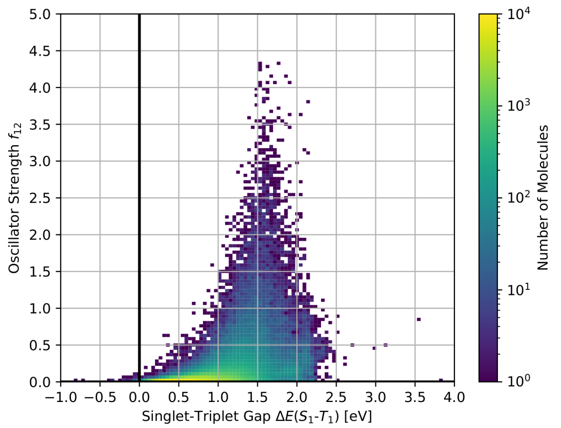


B. Vertical excitation energy and singlet-triplet gap.

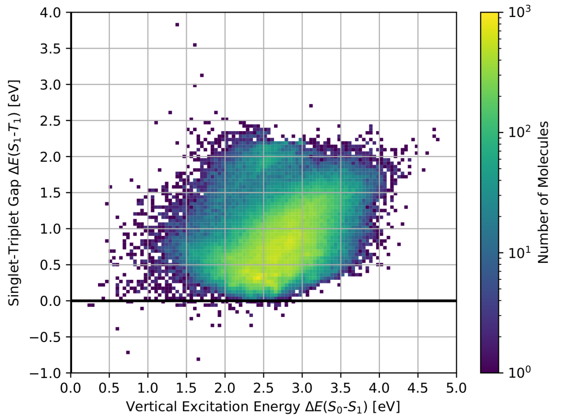


C. Vertical excitation energy and oscillator strength.

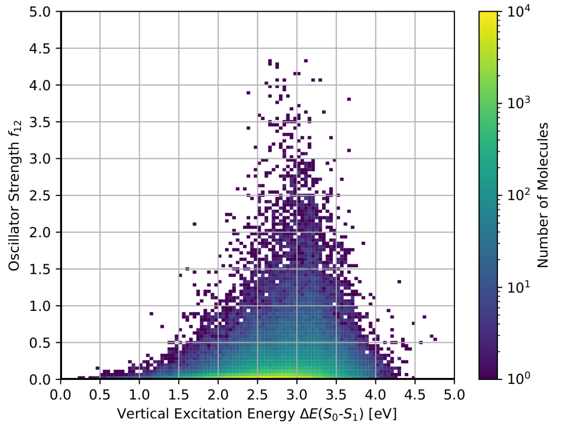


**Supplementary Figure 6.** Property distributions of all the compounds generated during experiment 4 of the artificial design stage at the ωB2PLYP' level of theory colored by the number of molecules in the respective property windows (A-C).

A. Singlet-triplet gap and oscillator strength.

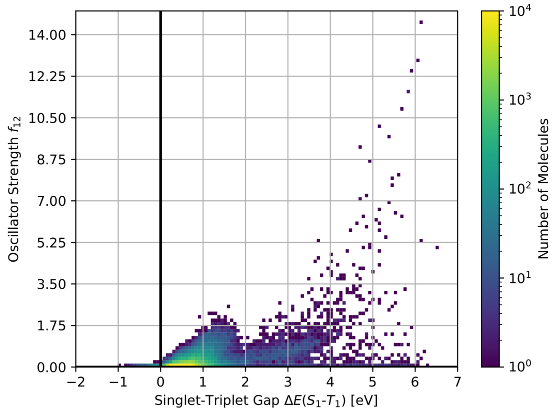


B. Vertical excitation energy and singlet-triplet gap.

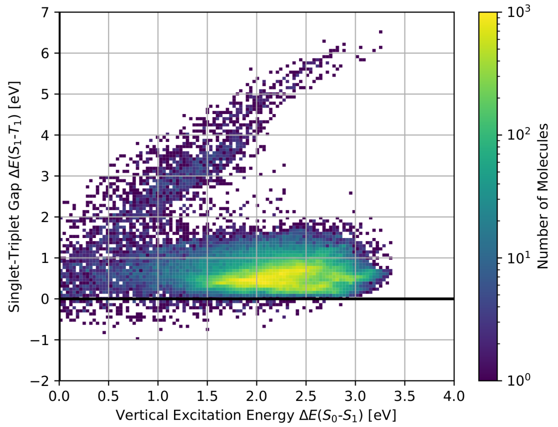


C. Vertical excitation energy and oscillator strength.

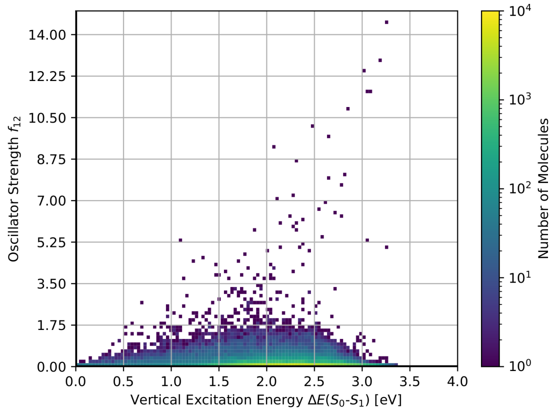


**Supplementary Figure 7.** Property distributions of all the compounds generated during experiment 5 of the artificial design stage at the ωB2PLYP' level of theory colored by the number of molecules in the respective property windows (A-C).

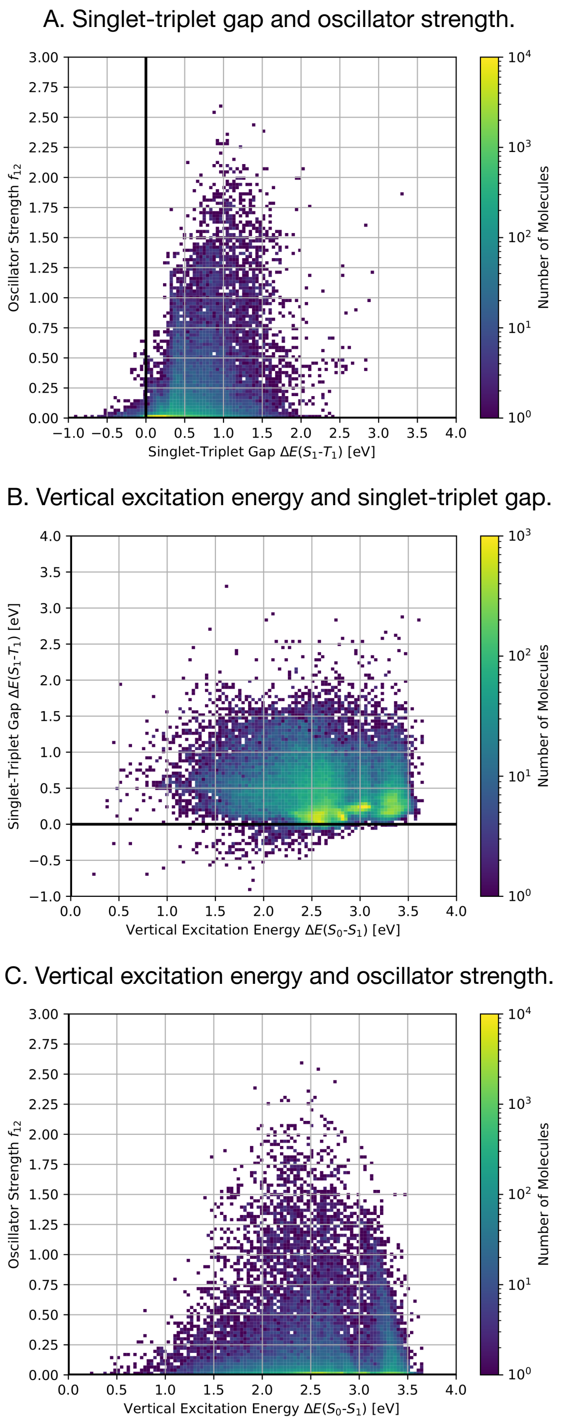


**Supplementary Figure 8.** Property distributions of all the compounds generated during experiment 6 of the artificial design stage at the ωB2PLYP’ level of theory colored by the number of molecules in the respective property windows (A-C).

Finally, as detailed in the main text, we generated both profactuals and counterfactuals using the model agnostic counterfactual compounds with STONED (MACCS) workflow. The corresponding results are depicted in Supplementary Figures 9-18.

A. Profactual compounds.

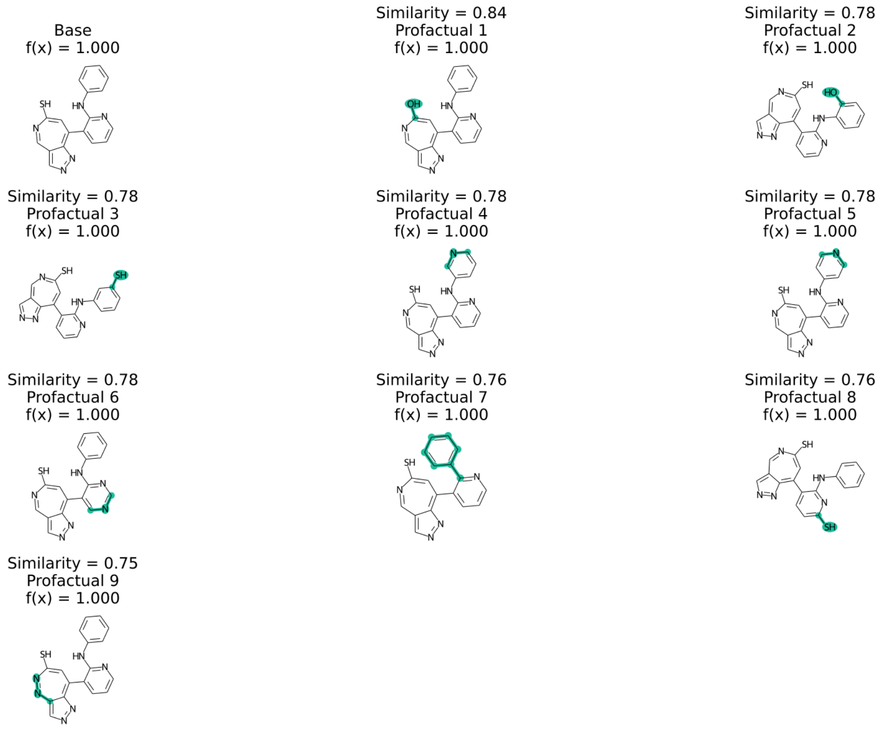


B. Counterfactual compounds.

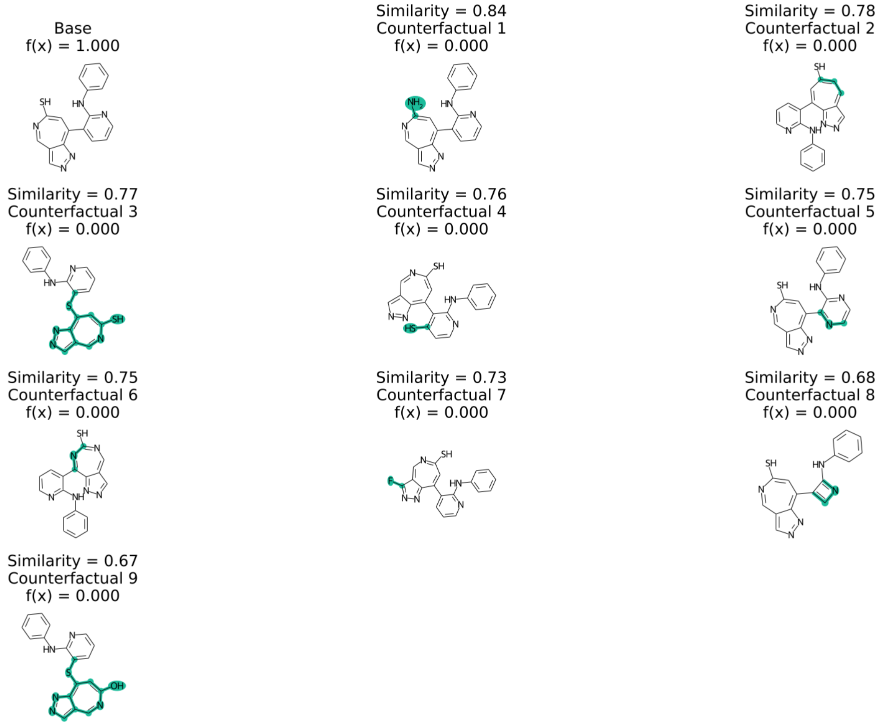


**Supplementary Figure 9.** Profactual (A) and counterfactual (B) compounds explaining the predictions of the artificial neural network classifier trained in artificial design experiment 2 using compound **4** as baseline.

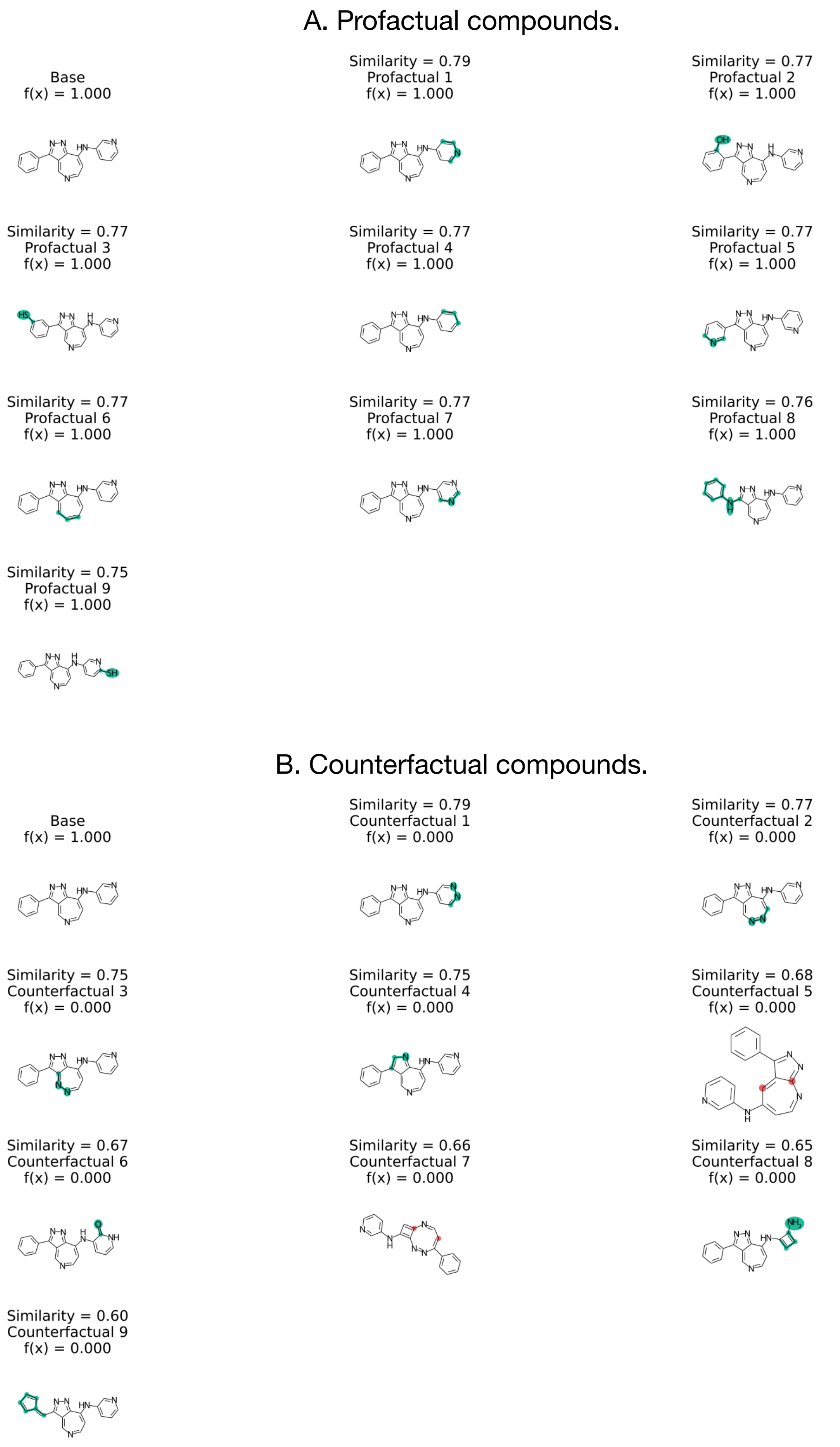


**Supplementary Figure 10.** Profactual (A) and counterfactual (B) compounds explaining the predictions of the artificial neural network classifier trained in artificial design experiment 2 using compound **5** as baseline.

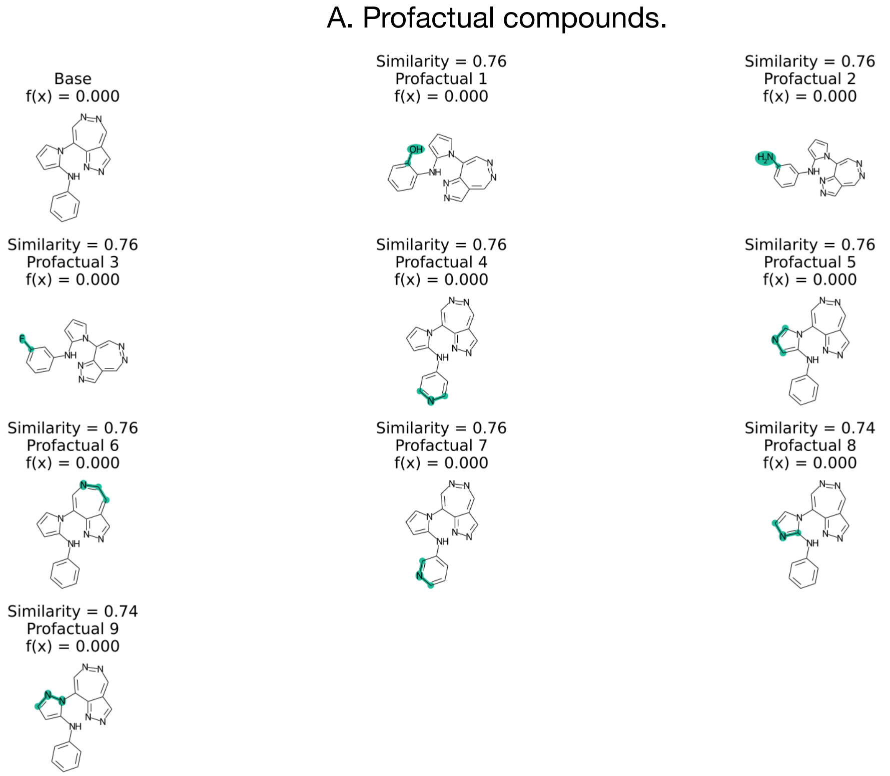


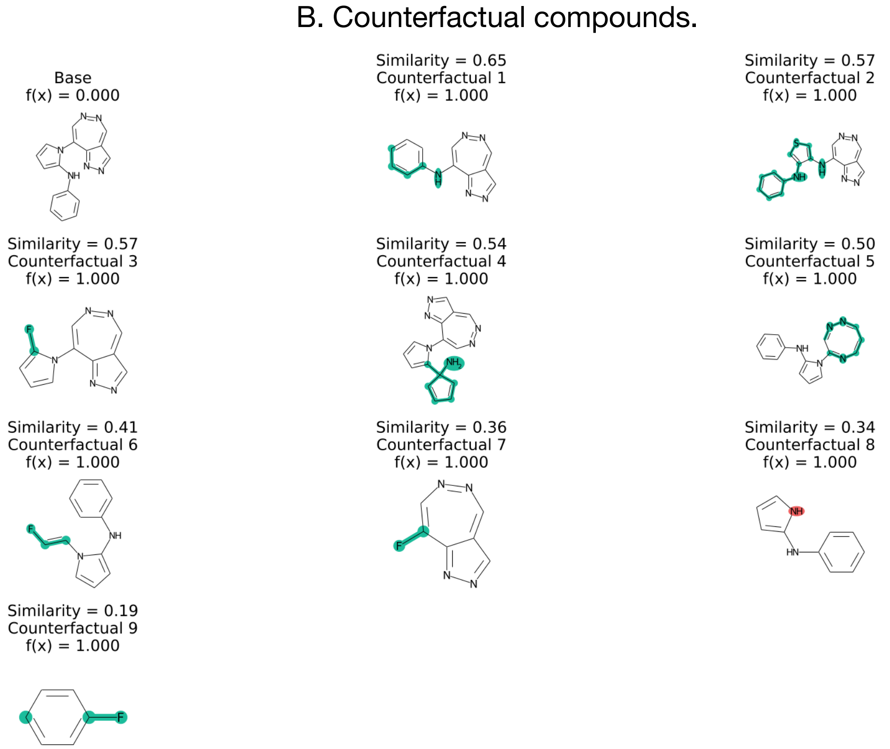


**Supplementary Figure 11.** Profactual (A) and counterfactual (B) compounds explaining the predictions of the artificial neural network classifier trained in artificial design experiment 3 using compound **6** as baseline.

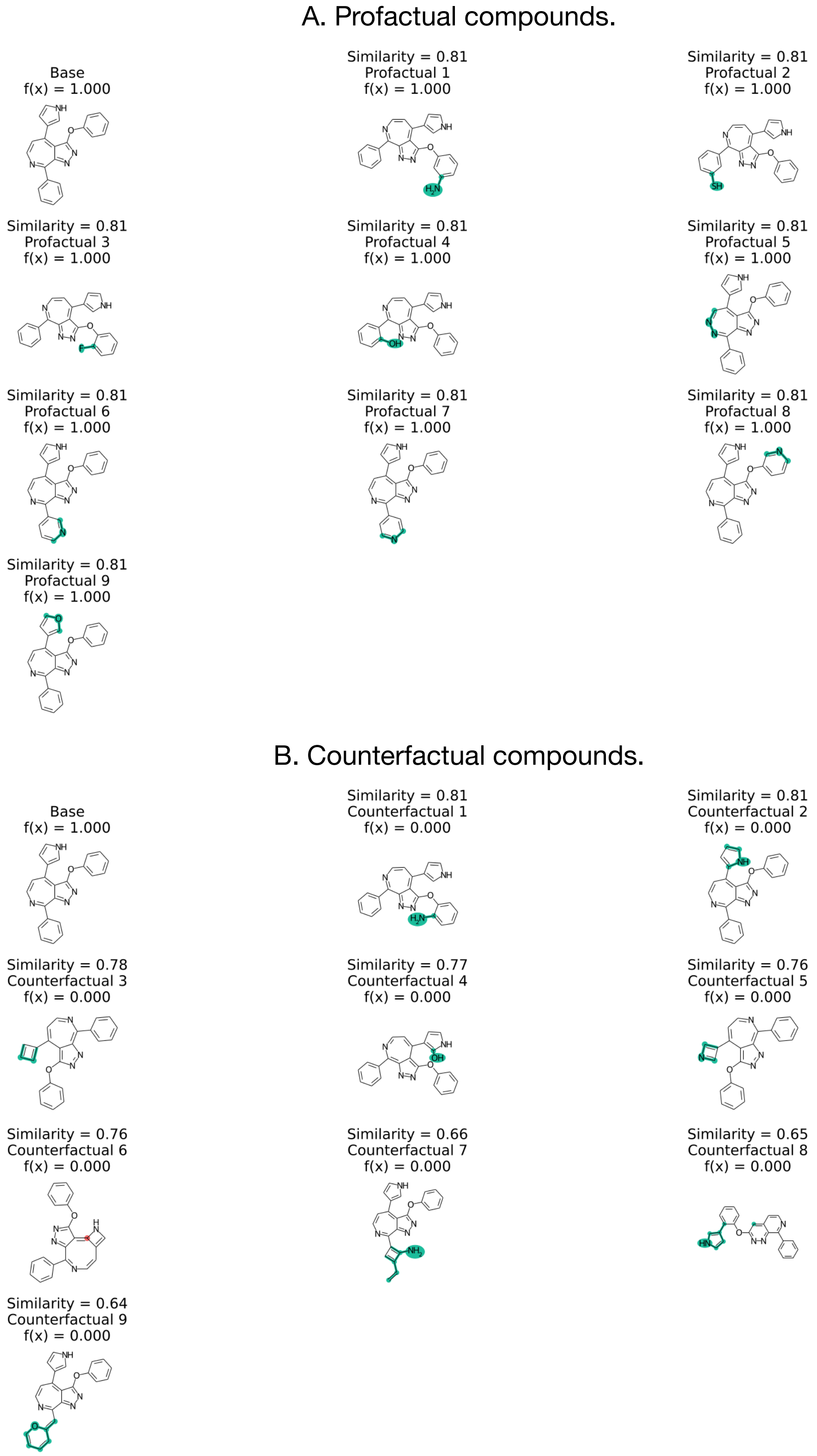


**Supplementary Figure 12.** Profactual (A) and counterfactual (B) compounds explaining the predictions of the artificial neural network classifier trained in artificial design experiment 3 using compound **7** as baseline.

A. Profactual compounds.

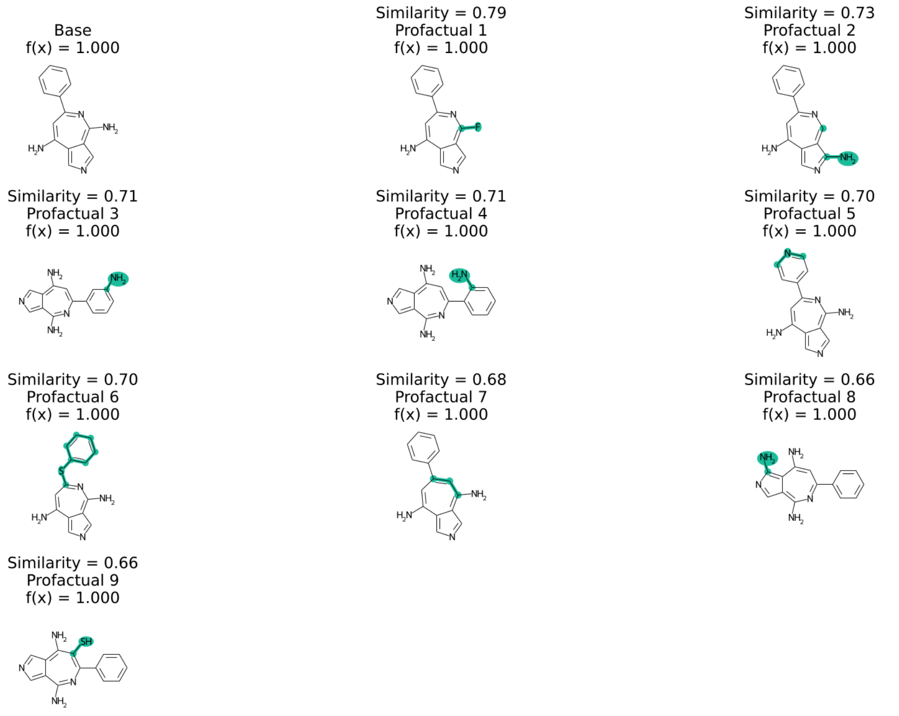


B. Counterfactual compounds.

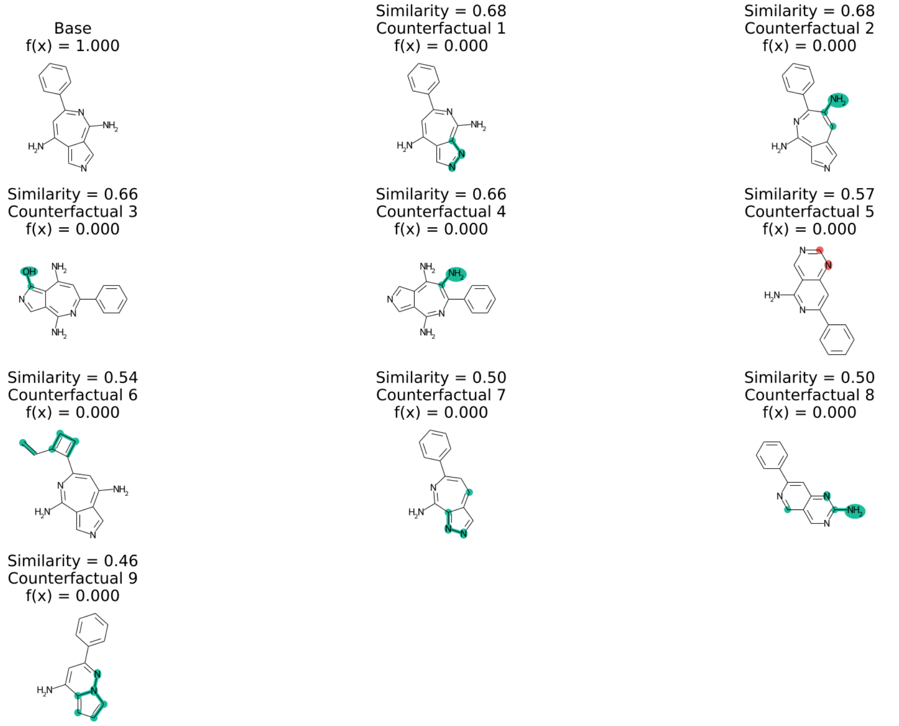


**Supplementary Figure 13.** Profactual (A) and counterfactual (B) compounds explaining the predictions of the artificial neural network classifier trained in artificial design experiment 4 using compound **8** as baseline.

A. Profactual compounds.

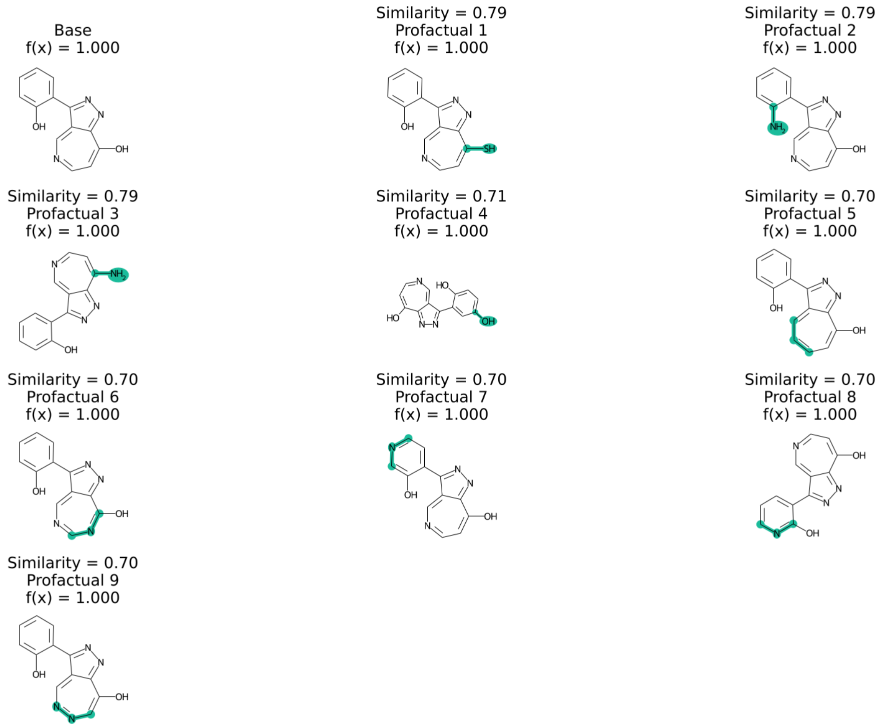


B. Counterfactual compounds.

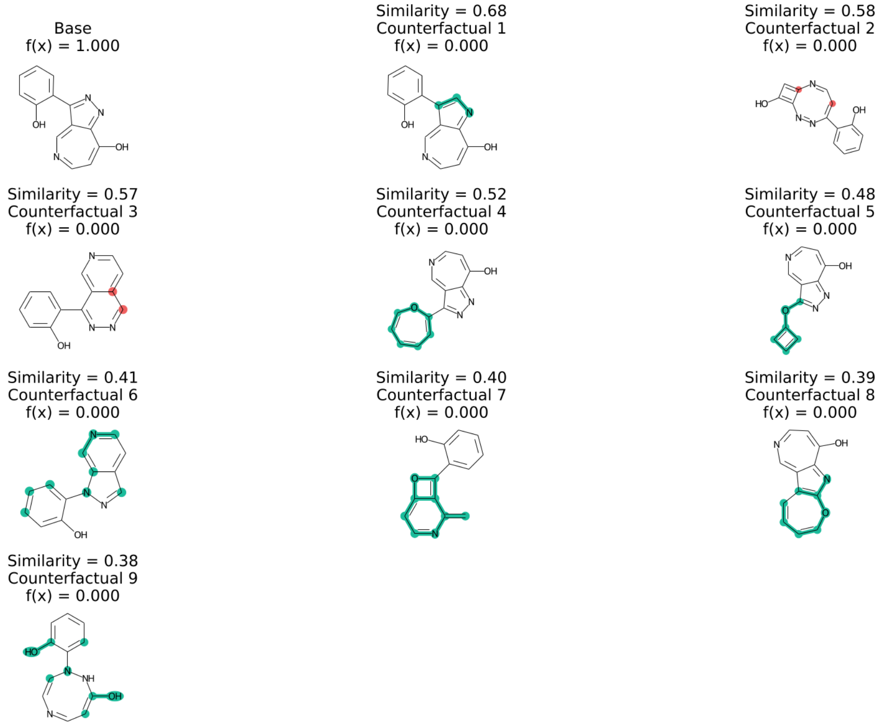


**Supplementary Figure 14.** Profactual (A) and counterfactual (B) compounds explaining the predictions of the artificial neural network classifier trained in artificial design experiment 4 using compound **9** as baseline.

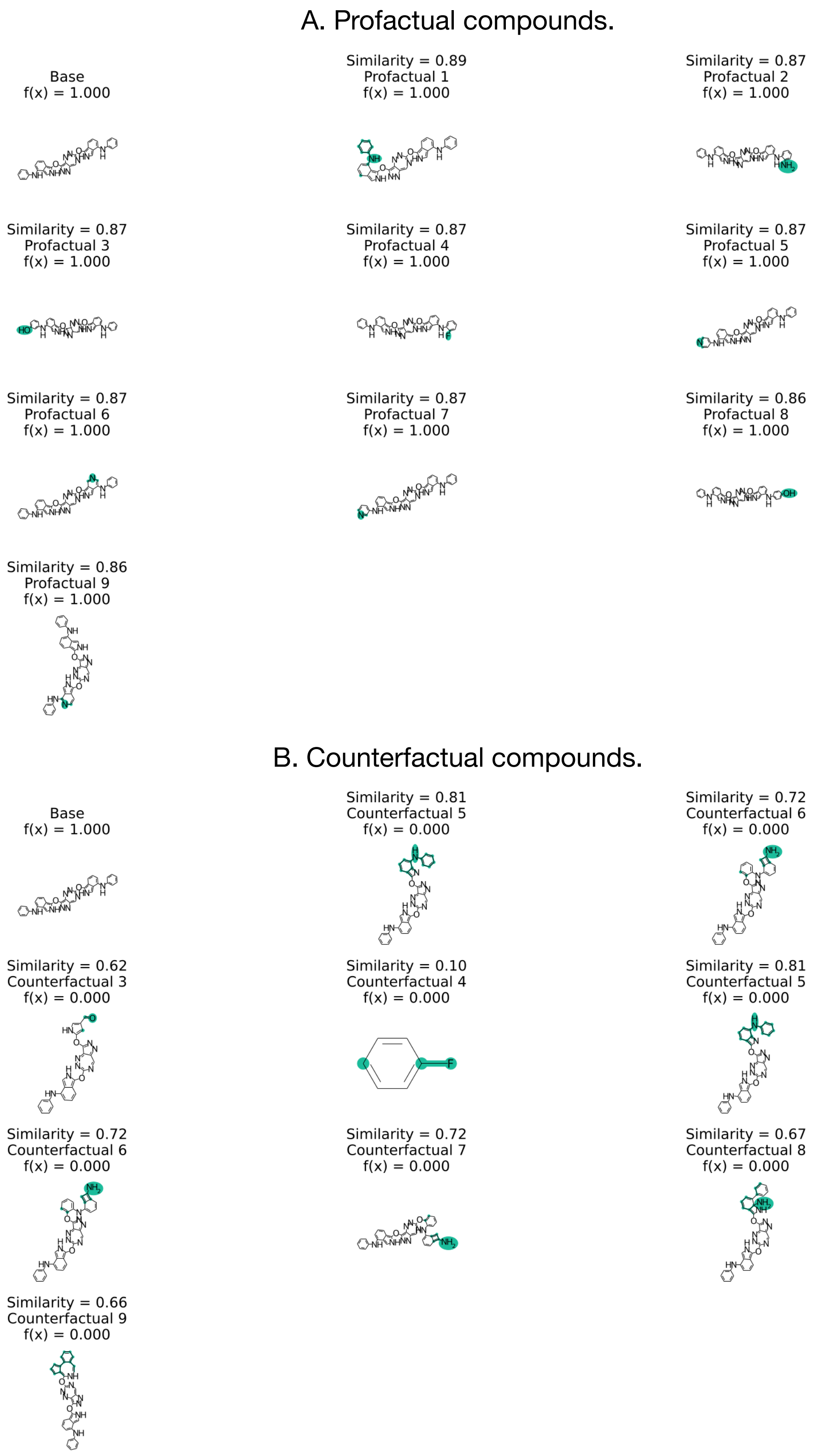


**Supplementary Figure 15.** Profactual (A) and counterfactual (B) compounds explaining the predictions of the artificial neural network classifier trained in artificial design experiment 5 using compound **10** as baseline.

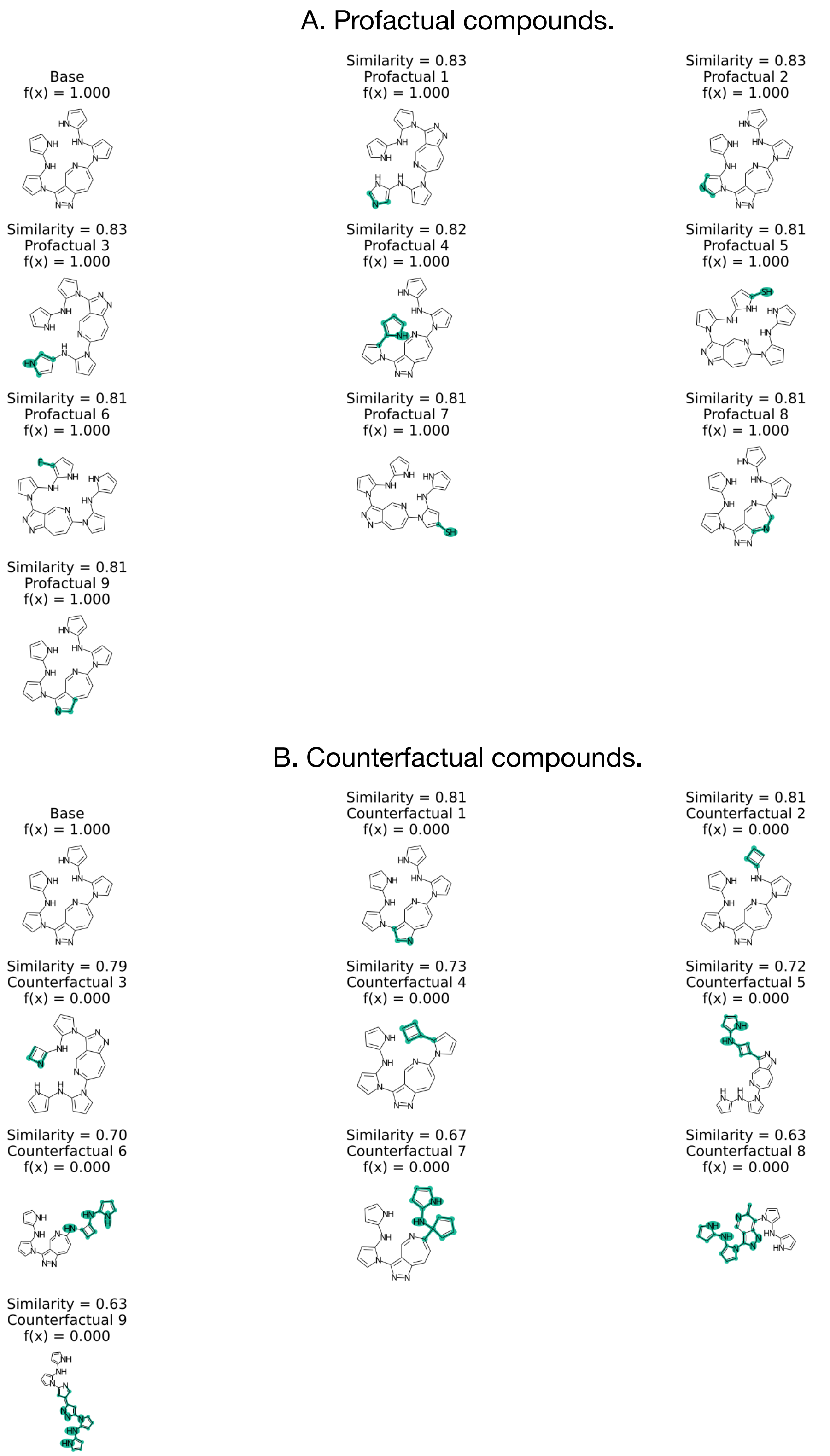


**Supplementary Figure 16.** Profactual (A) and counterfactual (B) compounds explaining the predictions of the artificial neural network classifier trained in artificial design experiment 5 using compound **11** as baseline.

A. Profactual compounds.

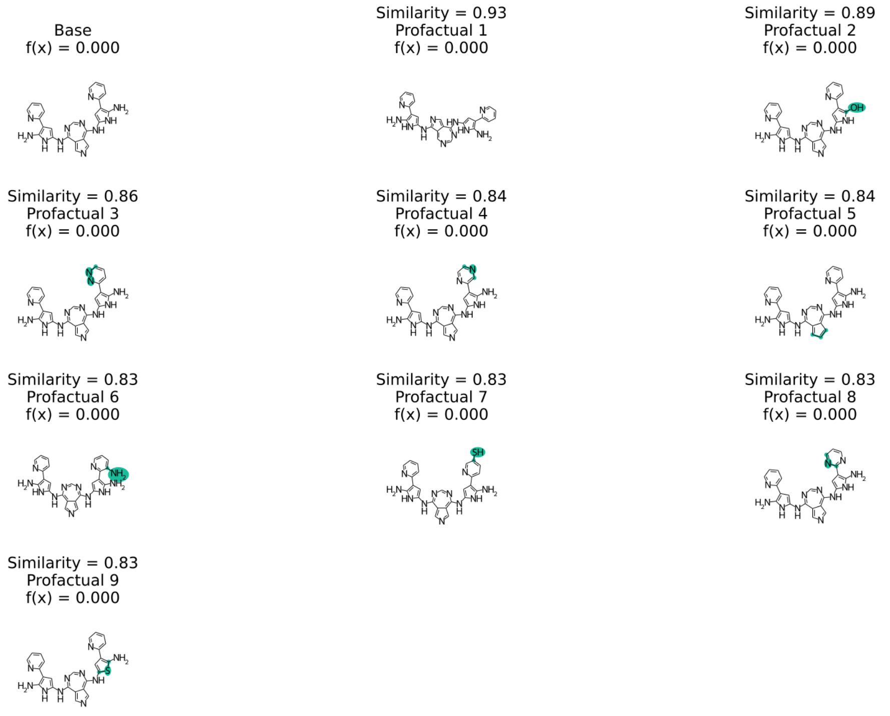


B. Counterfactual compounds.

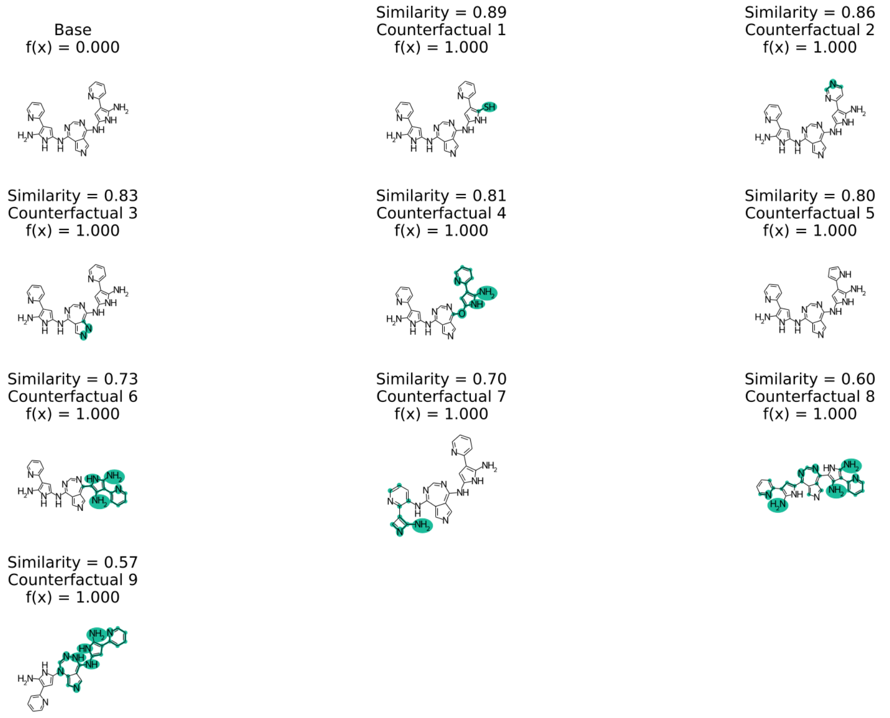


**Supplementary Figure 17.** Profactual (A) and counterfactual (B) compounds explaining the predictions of the artificial neural network classifier trained in artificial design experiment 6 using compound **12** as baseline.

## A. Profactual compounds.

Base
f(x) = 1.000

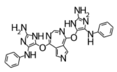

Similarity = 0.93
Profactual 1
f(x) = 1.000

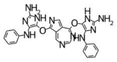

Similarity = 0.90
Profactual 2
f(x) = 1.000

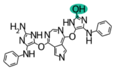

Similarity = 0.85
Profactual 3
f(x) = 1.000

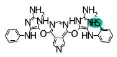

Similarity = 0.85
Profactual 4
f(x) = 1.000

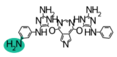

Similarity = 0.85
Profactual 5
f(x) = 1.000

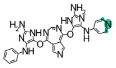

Similarity = 0.85
Profactual 6
f(x) = 1.000

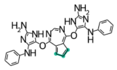

Similarity = 0.84
Profactual 7
f(x) = 1.000

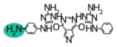

Similarity = 0.84
Profactual 8
f(x) = 1.000

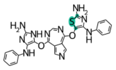

Similarity = 0.82
Profactual 9
f(x) = 1.000

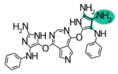

## B. Counterfactual compounds.

Base
f(x) = 1.000

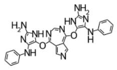

Similarity = 0.84
Counterfactual 9
f(x) = 0.000

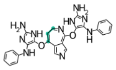

Similarity = 0.77
Counterfactual 2
f(x) = 0.000

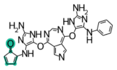

Similarity = 0.67
Counterfactual 3
f(x) = 0.000

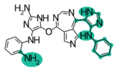

Similarity = 0.65
Counterfactual 4
f(x) = 0.000

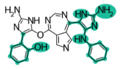

Similarity = 0.65
Counterfactual 5
f(x) = 0.000

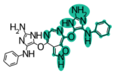

Similarity = 0.59
Counterfactual 6
f(x) = 0.000

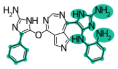

Similarity = 0.59
Counterfactual 7
f(x) = 0.000

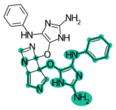

Similarity = 0.44
Counterfactual 8
f(x) = 0.000

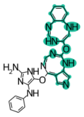

Similarity = 0.84
Counterfactual 9
f(x) = 0.000

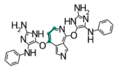

**Supplementary Figure 18.** Profactual (A) and counterfactual (B) compounds explaining the predictions of the artificial neural network classifier trained in artificial design experiment 6 using compound **13** as baseline.

**Lead Validation.** A comprehensive comparison of the simulated properties for the structures in the validation dataset at the SOS-ADC(2)/cc-pVDZ level of theory against the corresponding results at the ωB2PLYP'/def2-mSVP level of theory is depicted in Supplementary Figure 19. The linear regression results in Supplementary Table 3 show that while vertical excitation energies from both levels of theory show excellent agreement, both oscillator strength and singlet-triplet gaps compare less favorably. Nevertheless, for both properties, trends are reproduced reasonably well which demonstrates that ωB2PLYP'/def2-mSVP is appropriate to evaluate the fitness of the most promising candidates in the artificial design stage.

**Supplementary Table 3.** Linear regression results of simulated excited state properties for the validation set compounds at the ωB2PLYP' level of theory against SOS-ADC(2) properties: Y = Slope · X + Intercept.

| **Method** | **Property** | **Slope** | **Intercept** | **$R^2$** | **F** | **N** |
|---|---|---|---|---|---|---|
| SOS-ADC(2) | $\Delta E(S_0\text{-}S_1)$ | 0.926(1) | -0.129(3) | 0.98 | $8.27 \cdot 10^5$ | 13201 |
| SOS-ADC(2) | $\Delta E(S_1\text{-}T_1)$ | 0.510(4) | -0.148(3) | 0.53 | $1.49 \cdot 10^4$ | 13201 |
| SOS-ADC(2) | $f_{12}$ | 0.546(3) | -0.002(1) | 0.66 | $2.46 \cdot 10^4$ | 13201 |

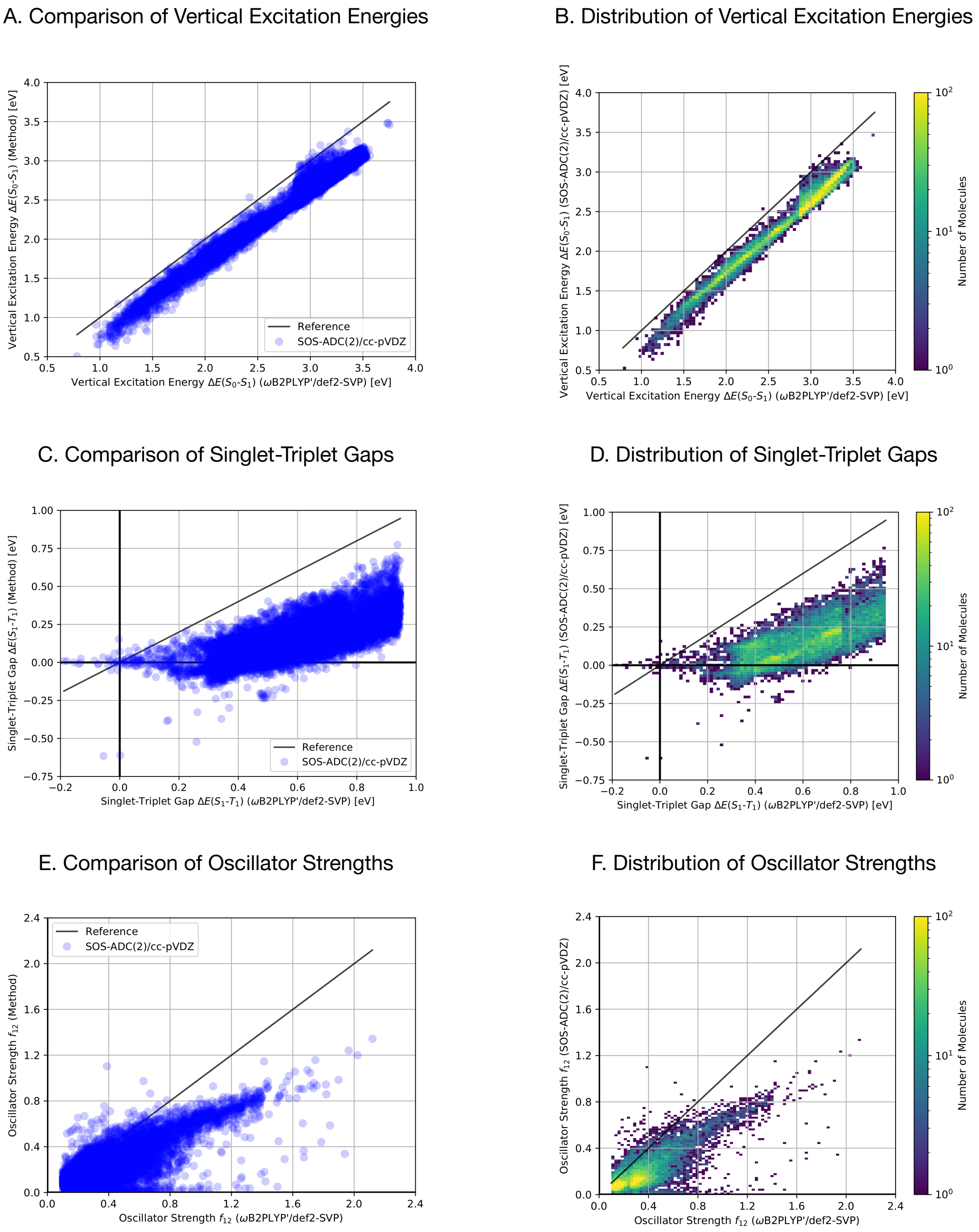


**Supplementary Figure 19.** Comparison of simulated excited state properties for the validation set compounds at the ωB2PLYP' and the SOS-ADC(2) levels of theory. A. Comparison of vertical excitation energies. B. Distribution of the comparison of vertical excitation energies. C. Comparison of singlet-triplet gaps. D. Distribution of the comparison of singlet-triplet gaps. E. Comparison of oscillator strengths. F. Distribution of the comparison of oscillator strengths.

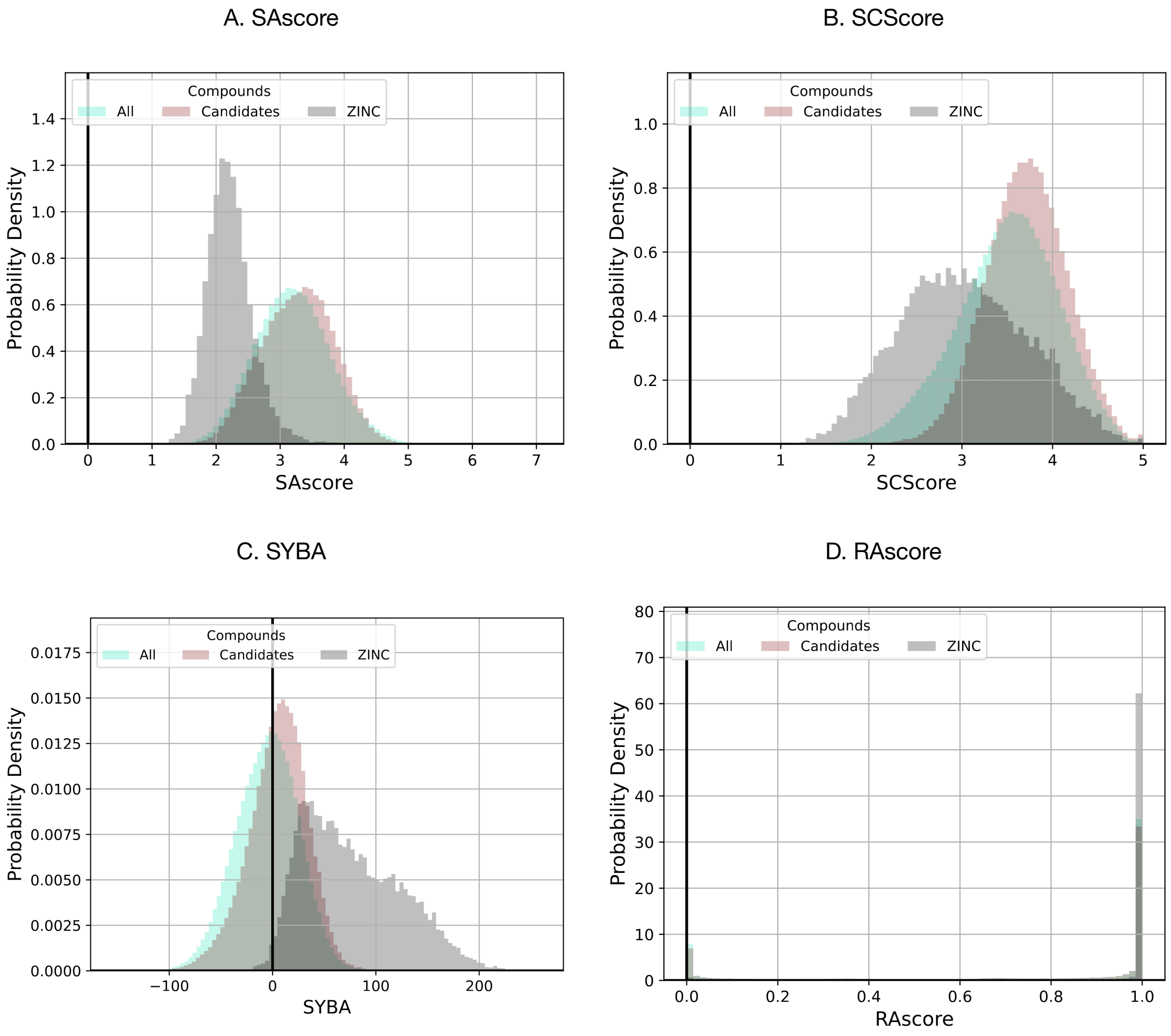


**Supplementary Figure 20.** Comparison of histograms of four distinct synthesizability metrics between all compounds proposed during the artificial design stage and the subset of candidates that is estimated to consist of INVEST compounds against a subset generated based on the ZINC20 dataset.

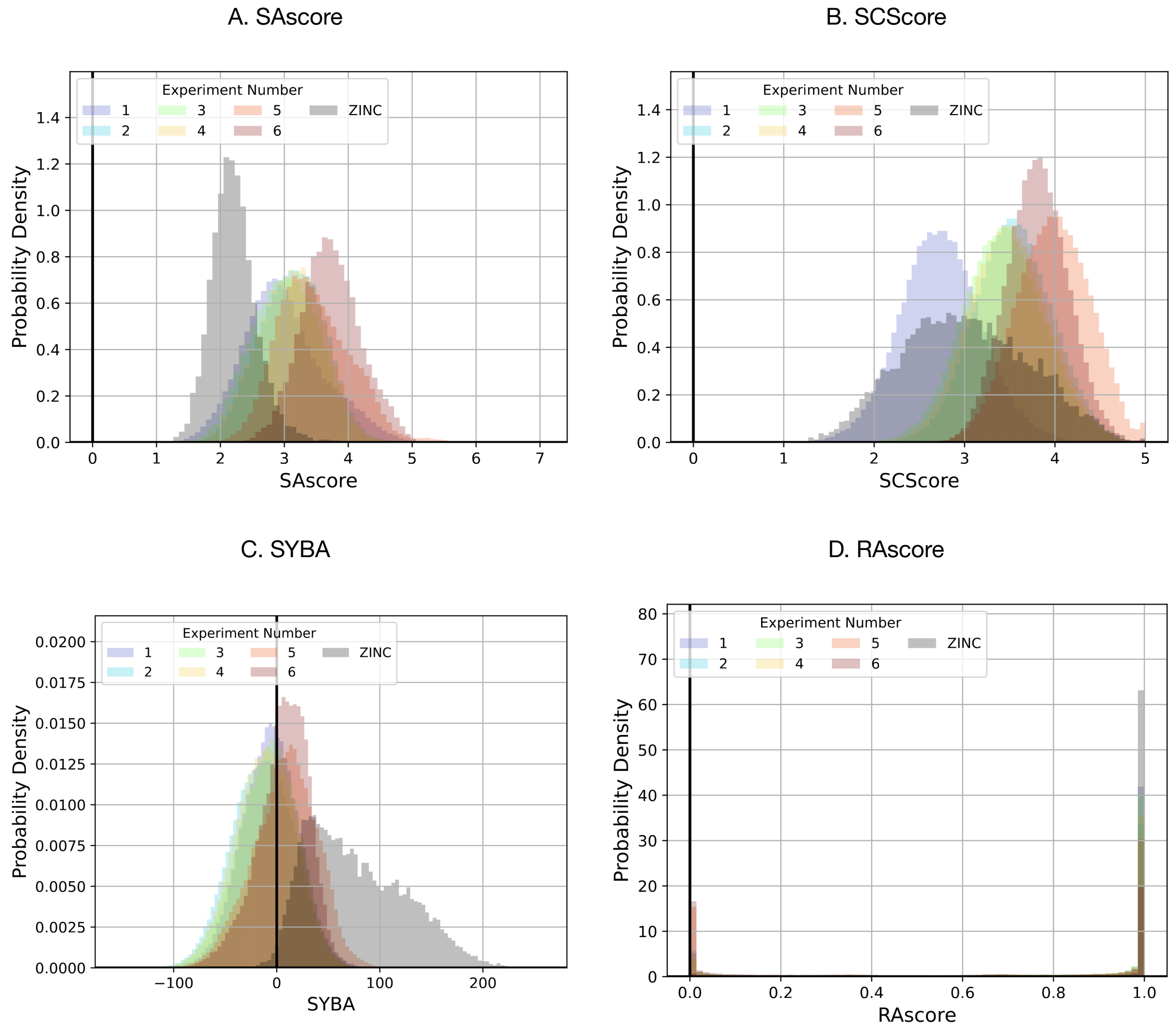


**Supplementary Figure 21.** Comparison of histograms of four distinct synthesizability metrics between the six different artificial design experiments conducted against a subset generated based on the ZINC20 dataset.

## References


(1) Irwin, J. J.; Tang, K. G.; Young, J.; Dandarchuluun, C.; Wong, B. R.; Khurelbaatar, M.; Moroz, Y. S.; Mayfield, J.; Sayle, R. A. ZINC20—A Free Ultralarge-Scale Chemical Database for Ligand Discovery. *J. Chem. Inf. Model.* **2020**, *60* (12), 6065–6073. https://doi.org/10.1021/acs.jcim.0c00675.

(2) Akiba, T.; Sano, S.; Yanase, T.; Ohta, T.; Koyama, M. Optuna: A next-Generation Hyperparameter Optimization Framework. In *Proceedings of the 25th ACM SIGKDD international conference on knowledge discovery & data mining*; KDD ’19; Association for Computing Machinery: New York, NY, USA, 2019; pp 2623–2631. https://doi.org/10.1145/3292500.3330701.

(3) Wang, M.; Zheng, D.; Ye, Z.; Gan, Q.; Li, M.; Song, X.; Zhou, J.; Ma, C.; Yu, L.; Gai, Y.; Xiao, T.; He, T.; Karypis, G.; Li, J.; Zhang, Z. Deep Graph Library: A Graph-Centric, Highly-Performant Package for Graph Neural Networks. *arXiv preprint arXiv:1909.01315* **2019**.
(4) Rogers, D.; Hahn, M. Extended-Connectivity Fingerprints. *J. Chem. Inf. Model.* **2010**, *50* (5), 742–754. https://doi.org/10.1021/ci100050t.
(5) Pracht, P.; Grimme, S. *Conformer-Rotamer Ensemble Sampling Tool.* https://github.com/grimme-lab/crest (accessed 2020-06-04).
(6) Grimme, S. Exploration of Chemical Compound, Conformer, and Reaction Space with Meta-Dynamics Simulations Based on Tight-Binding Quantum Chemical Calculations. *J. Chem. Theory Comput.* **2019**, *15* (5), 2847–2862. https://doi.org/10.1021/acs.jctc.9b00143.
(7) Pracht, P.; Bohle, F.; Grimme, S. Automated Exploration of the Low-Energy Chemical Space with Fast Quantum Chemical Methods. *Phys. Chem. Chem. Phys.* **2020**, *22* (14), 7169–7192. https://doi.org/10.1039/C9CP06869D.
(8) Spicher, S.; Grimme, S. Robust Atomistic Modeling of Materials, Organometallic, and Biochemical Systems. *Angew. Chem. Int. Ed.* **2020**, *59* (36), 15665–15673. https://doi.org/10.1002/anie.202004239.
(9) Spicher, S.; Grimme, S. Efficient Computation of Free Energy Contributions for Association Reactions of Large Molecules. *J. Phys. Chem. Lett.* **2020**, *11* (16), 6606–6611. https://doi.org/10.1021/acs.jpclett.0c01930.
(10) Pracht, P.; Grant, D. F.; Grimme, S. Comprehensive Assessment of GFN Tight-Binding and Composite Density Functional Theory Methods for Calculating Gas-Phase Infrared Spectra. *J. Chem. Theory Comput.* **2020**, *16* (11), 7044–7060. https://doi.org/10.1021/acs.jctc.0c00877.
(11) Grimme, S. *Semiempirical Extended Tight-Binding Program Package*. https://github.com/grimme-lab/xtb (accessed 2020-06-04).
(12) Grimme, S.; Bannwarth, C.; Shushkov, P. A Robust and Accurate Tight-Binding Quantum Chemical Method for Structures, Vibrational Frequencies, and Noncovalent Interactions of Large Molecular Systems Parametrized for All Spd-Block Elements (Z = 1–86). *J. Chem. Theory Comput.* **2017**, *13* (5), 1989–2009. https://doi.org/10.1021/acs.jctc.7b00118.
(13) Bannwarth, C.; Ehlert, S.; Grimme, S. GFN2-xTB—An Accurate and Broadly Parametrized Self-Consistent Tight-Binding Quantum Chemical Method with Multipole Electrostatics and Density-Dependent Dispersion Contributions. *J. Chem. Theory Comput.* **2019**, *15* (3), 1652–1671. https://doi.org/10.1021/acs.jctc.8b01176.
(14) Yang, K.; Swanson, K.; Jin, W.; Coley, C.; Eiden, P.; Gao, H.; Guzman-Perez, A.; Hopper, T.; Kelley, B.; Mathea, M.; Palmer, A.; Settels, V.; Jaakkola, T.; Jensen, K.; Barzilay, R. Analyzing Learned Molecular Representations for Property Prediction. *J. Chem. Inf. Model.* **2019**, *59* (8), 3370–3388. https://doi.org/10.1021/acs.jcim.9b00237.
